# Deepfake: definitions, performance metrics and standards, datasets, and a meta-review

**DOI:** 10.3389/fdata.2024.1400024

**Published:** 2024-09-04

**Authors:** Enes Altuncu, Virginia N. L. Franqueira, Shujun Li

**Affiliations:** Institute of Cyber Security for Society (iCSS) & School of Computing, University of Kent, Canterbury, United Kingdom

**Keywords:** deepfake, survey, definition, datasets, standards, performance metrics

## Abstract

Recent advancements in AI, especially deep learning, have contributed to a significant increase in the creation of new realistic-looking synthetic media (video, image, and audio) and manipulation of existing media, which has led to the creation of the new term “deepfake.” Based on both the research literature and resources in English, this paper gives a comprehensive overview of deepfake, covering multiple important aspects of this emerging concept, including (1) different definitions, (2) commonly used performance metrics and standards, and (3) deepfake-related datasets. In addition, the paper also reports a meta-review of 15 selected deepfake-related survey papers published since 2020, focusing not only on the mentioned aspects but also on the analysis of key challenges and recommendations. We believe that this paper is the most comprehensive review of deepfake in terms of the aspects covered.

## 1 Introduction

Recent advancements in AI and machine learning have increased the capability to produce more realistic media, e.g., video, image, and audio. Especially, state-of-the-art deep learning methods enabled the generation of “deepfakes,” manipulated or synthetic media the realness of which are not easily recognisable by the human eye. Although deepfake is a relatively new phenomenon (having first appeared at the end of 2017), its growth has been remarkable. According to the 2019 and 2020 Deeptrace (now, Sensity) reports on the state of deepfake (Ajder et al., 2019), the number of deepfake videos on the English-speaking internet grew from 7,964 (December 2018) to 14,678 (July 2019) to 85,047 (December 2020), representing a 968% increase from 2018 to 2020. By 2024, the number of available tools for deepfake generation has reached to over 10,000 (Sensity, 2024).

In this work, we review the existing deepfake-related research ecosystem in terms of various aspects, including performance metrics, standards, and datasets. Furthermore, we provide a meta-review of 15 selected deepfake-related survey papers which covers several additional aspects other than the mentioned ones in a systematic manner, such as performance comparison, key challenges, and recommendations.

Despite being a hugely popular term, there is a lack of consensus on the definition of “deepfake” and the boundary between deepfakes and non-deepfakes is not clear cut. For this survey, we adopt a relatively more inclusive approach to cover all forms of manipulated or synthetic media that are considered deepfakes in a broader sense. We also cover closely related topics including biometrics and multimedia forensics, since deepfakes are often used to launch presentation attacks against biometrics-based authentication systems and detection of deepfakes can be considered part of multimedia forensics. A more detailed discussion on different definitions of “deepfake” is given next.

### 1.1 Definitions of the term deepfake

As its name implies, the term “deepfake” is derived from the combination of “deep” [referring to *deep learning* (DL)] and “fake.” It is normally used to refer to the manipulation of existing media (image, video, and/or audio) or the generation of new (synthetic) media using DL-based approaches. The most commonly discussed deepfake data are fake face images, fake speech forgeries, and fake videos that combine both fake images and fake speech forgeries. While having “fake” in the word indicates manipulated or synthesised media, there are plenty of benign applications of the deepfake technology, e.g., for entertainment and creative arts. With this respect, another term “deep synthesis” has been proposed as a more neutral-sounding alternative (Tencent, 2020). This new term, however, has not been widely adopted.

In addition to the lack of a universal definition, as mentioned already, the boundary between deepfakes and non-deep fakes is actually not clear-cut. There are at least two important aspects we should consider, one on detection of and the other on creation of deepfakes.

First, the detection of deepfakes often follows very similar approaches to the detection of traditional fakes generated without using DL techniques. Advanced detection methods have also started leveraging DL to improve their performance, but they do not necessarily need to know how a target media is created (deep or not). To some extent, one could argue that detecting deepfakes does not involve developing deepfake-specific methods (even though some researchers choose to do so), but a more robust and universal detector that can handle any (deep or not) fake media. This can be seen in two closely related topics: biometrics and multimedia forensics. For biometrics, there is a trend of using deep learning techniques to generate fake biometric signals (e.g., face images and videos) for biometric spoofing or presentation attacks. For multimedia forensics, deepfake-based forgeries have become a new threat to the traditional problem of “forgery detection.” For both topics, the detection of biometric spoofing and multimedia forgeries have evolved to consider both deep and non-deep fakes.

Second, one may argue that the word “deep” in “deepfake” does not necessarily refer to the use of “deep learning”, but any “deep” (i.e., sophisticated) technology that creates a very believable fake media. For instance, Brady (2020) considered deepfake as audio-visual manipulation using “a spectrum of technical sophistication ... and techniques.” They also introduced two new terms, *Shallowfake* and *Cheapfake*, referring to “low-level manipulation of audio-visual media created with (easily) accessible software [or no software] to speed, slow, restage or re-contextualise content.” This broader understanding of “deepfake” has also been adopted by lawmakers for new legislations combating malicious deepfakes. For instance, the following two United States acts define “deepfakes” as follows:

- 2018 Malicious Deep Fake Prohibition Act^1^:
  §1041.(b).(2): “*the term ‘deep fake' means an audiovisual record created or altered in a manner that the record would falsely appear to a reasonable observer to be an authentic record of the actual speech or conduct of an individual*.”
- 2019 DEEP FAKES Accountability Act^2^:
  §1041.(n).(3): “*The term ‘deep fake' means any video recording, motion-picture film, sound recording, electronic image, or photograph, or any technological representation of speech or conduct substantially derivative thereof*
*(A) which appears to authentically depict any speech or conduct of a person who did not in fact engage in such speech or conduct; and*
  *(B) the production of which was substantially dependent upon technical means, rather than the ability of another person to physically or verbally impersonate such person*.”

As we can see from the above legal definitions of “deepfake,” the use of DL as a technology is not mentioned at all. The focus here is on “authenticity”, “impersonation” and (any) “technical means.”

### 1.2 Scope and contribution

Based on the above discussion on definitions of deepfake, we can see it is not always straightforward or meaningful to differentiate deepfakes from non-deep fakes. In addition, for our focus on performance evaluation and comparison, the boundary between deepfakes and non-deep fakes is even more blurred. This is because DL is just a special (deeper) form of machine learning (ML), and as a result, DL and non-deep ML methods share many common concepts, metrics and procedures.

Despite the fact that deepfake may be understood in a much broader sense, in this work, we have a sufficiently narrower focus to avoid covering too many topics. We, therefore, decided to define the scope of this survey as follows:

- For metrics and standards, we chose to include all commonly used ones for evaluating general ML methods and those specifically defined for evaluating deepfake creation or detection methods.
- For datasets, we considered those related to fake media covered in the deepfake-related survey papers and those with an explicit mention of the term “deepfake” or a comparable term.
- For the meta-review, we considered only survey papers whose authors explicitly referred to the term “deepfakes” in the metadata (title, abstract, and keywords).

In this paper, we aim to make the following contributions:

- We discuss existing definitions of the term “deepfake” and propose a more inclusive definition.
- We present an overview of the available deepfake-related standards and metrics for evaluating deepfake generation or detection, which have been generally overlooked by previous surveys. The covered metrics include general AI metrics as well as several deepfake-specific metrics for objective and subjective evaluation.
- We comprehensively cover a wide range of deepfake datasets, considering different modalities—image, video, audio, and text. We believe this paper offers the most comprehensive review of deepfake-related datasets so far.
- We provide a meta-review of 15 deepfake survey papers to draw some high-level insights for monitoring the future development of deepfake-related technologies and their applications.

### 1.3 Paper organisation

The rest of the paper is as organised as follows. In Section 2, we mention how we collected the survey papers covered in this paper. Then, Section 3 reviews existing deepfake-related performance metrics and standards, followed by Section 4 covering deepfake datasets. In Section 5, we provide a meta-review of the survey papers collected. Finally, the paper concludes with Section 6.

## 2 Methodology

Research papers covered in this survey (i.e., the deepfake-related survey papers) were identified via systematic searches on the Scopus scientific database. The following search query was used to perform the searches on Scopus:

(deepfake* OR deep-fake* OR “deep fake*”) AND (review OR survey OR overview OR systemati* OR SoK)

The searches returned 117 survey papers in English, published between 2020 and 2024 (inclusive). Out of these papers, 15 papers were selected for consideration in the meta-review. During the selection process, all the papers were carefully reviewed, and only the ones having a substantial comparative angle, e.g., those with performance comparison and/or covering different datasets, tools, challenges, competitions, metrics, etc., were included. Furthermore, for the papers with similar coverage, those published in more decent venues (e.g., higher-ranked journals or more well-known conferences) and/or more cited by other studies were preferred. Finally, we ensured that the final set of papers cover publications from each year between 2020 and 2024.

Deepfake-related datasets were compiled based on the selected survey papers and identified deepfake-related challenges, competitions, and benchmarks. Relevant standards were identified mainly via research papers covered in this survey, the co-authors' personal knowledge, and Google Web searches. For performance metrics, we covered those commonly used based on relevant standards, the survey papers, and the identified challenges, competitions, and benchmarks.

## 3 Deepfake-related performance metrics and standards

In this survey, we focus on performance evaluation and comparison of deepfake generation and detection methods. The metrics used for such performance evaluations are at the core of our discussions. In this section, we review the performance metrics that are commonly used to evaluate deepfake generation and detection algorithms. Note that all metrics covered in this section are also commonly used for evaluating the performance of similar systems that are not for generating or detecting deepfakes. Therefore, this section can be seen as a very brief tutorial on general performance metrics.

In the last subsection, we also briefly discuss how the related performance metrics are covered in formal standards. By “formal standards,” we refer to standards defined following a formal procedure, often by one or more established standardisation bodies such as the International Organization for Standardization (ISO)^3^ and the International Electrotechnical Commission (IEC).^4^ Note that we consider a broad range of documents defined to be standards by standardisation bodies, e.g., International Telecommunication Union (ITU)^5^ recommendations and ISO technical reports (TRs).

### 3.1 The confusion matrix

Deepfake detection is primarily a binary classification problem. A binary classifier takes an input that is *actually positive* or *actually negative* and outputs a binary value denoting it to be *predicted positive* or *predicted negative*. For example, a deepfake detection system will take a suspected image as the input that may be *actually fake* or *actually real* and output *predicted fake* or *predicted real*.

A fundamental tool used in evaluating a binary classifier is the **confusion matrix** that summarises the success and failure of the classification model. On one axis are the two *actual* values and on the other axis are the two *predicted* values. The classification is *successful/correct/true* (true positive and true negative) when the actual and the predicted values match. It is *failed/incorrect/false* (false positive and false negative) when the actual and predicted values do not match. Table 1 shows the confusion matrix for a binary deepfake classifier (detector). The two cells in green, TP (the number of **true positives**) and TN (the number of **true negatives**), indicate correct prediction results, and the two cells in red, FN (the number of **false negatives**), and FP (the number of **false positives**), indicate two different types of errors when making incorrect prediction results.

**Table 1:** Confusion matrix for a binary classifier for detecting deepfake.

	**Fake (predicted)**	**Real (predicted)**
Fake (actual)	TP	FN
Real (actual)	FP	TN

### 3.2 Precision and recall

Based on the four fundamental values introduced in Section 3.1, i.e., TP, TN, FP, and FN, we define two important performance metrics for a binary classifier—**precision** and **recall**.

Precision of a binary classifier is defined as the fraction of *actually positive* samples among all the *predicted positives*. In the confusion matrix, it is the fraction of true samples in the first column. It can be formally defined as Equation 1.


(1) $$\begin{array}{l} \text{precision}=\frac{\text{TP}}{\text{TP}+\text{FP}} \end{array}$$

When the “natural” ratio between positive and negative samples is significantly different from the test set, it is often useful to adjust the weight of the false positives, which leads to the **weighted precision** (wP) defined in Equation 2, where α>0 is a weight determined by the ratio between the negative and positive samples.


(2) $$\begin{array}{l} \text{wP}=\frac{\text{TP}}{\text{TP}+\alpha \text{FP}} \end{array}$$

Recall of a binary classifier is the fraction of *predicted positive* samples among the *actually positive* samples, as shown in Equation 3. In the confusion matrix, it is the fraction of true samples in the first row.


(3) $$\begin{array}{l} \text{recall}=\frac{\text{TP}}{\text{TP}+\text{FN}} \end{array}$$

Let us consider an example binary classifier that predicts if an image from a database containing both deepfake and real (authentic) images is fake or not. Precision of the classifier is the fraction of correctly classified images among all images classified as deepfake. On the other hand, recall is the fraction of deepfake images identified by the classifier, among all deepfake images in the database.

### 3.3 True and false positive rates

Focusing on predicted positive samples, we can also define two metrics: **true positive rate** (TPR), also called **correct detection rate** (CDR), as the fraction of the predicted positive samples among the actually positive samples and **false positive rate** (FPR), also called **false alarm rate** (FAR), as the fraction of the predicted positive samples among the actually negative samples, as shown in Equations 4, 5. In the confusion matrix, TPR is the fraction of predicted positive samples in the first row and FPR is the fraction of predicted positive samples in the second row. Note that TPR is basically a different name for **recall** (Equation 3).


(4) $$\begin{array}{l} \text{TPR}=\frac{\text{TP}}{\text{TP}+\text{FN}} \end{array}$$

(5) $$\begin{array}{l} \text{FPR}=\frac{\text{FP}}{\text{FP}+\text{TN}} \end{array}$$

### 3.4 True and false negative rates

Similar to true and false positive rates, we can define two other rates focusing on negative predicted results: **true negative rate** (TNR) indicating the fraction of the predicted negative samples among the actually negative samples, and **false negative rate** (FNR) indicating the fraction of the predicted negative samples among the actually positive samples, as shown in Equations 6, 7.


(6) $$\begin{array}{l} \text{TNR}=\frac{\text{TN}}{\text{TN}+\text{FP}} \end{array}$$

(7) $$\begin{array}{l} \text{FNR}=\frac{\text{FN}}{\text{FN}+\text{TP}} \end{array}$$

### 3.5 Sensitivity and specificity

In some applications of binary classifiers, especially in biology and medicine, the TPR and the TNR are more commonly used, and they are often called **sensitivity** (TPR) and **specificity** (TNR). The focus of these two terms is on the two types of correctness of the predicted results. These are less used in deepfake-related research, hence, we will not refer to them in the remainder of this paper.

### 3.6 Equal error rate

Focusing on error rates means that we need to consider the FPR and the FNR. These two rates normally conflict with each other so that reducing one rate normally leads to an increase in the other. Therefore, rather than trying to reduce both error rates at the same time, which is normally impossible, the more realistic task in practical applications is to find the right balance so that they are both below an acceptable threshold.

In some applications, such as biometrics, people are particularly interested in establishing the so-called **equal error rate** (EER) or **crossover error rate** (CER), the point where the FPR and the FNR are equal. The EER/CER is not necessarily a good metric for some applications, especially when the two types of errors are of different levels of importance, e.g., for detecting critical deepfakes (e.g., fake news that can influence how people cast their votes) we can often tolerate more false positives (false alarms) than false negatives (missed alarms).

### 3.7 Accuracy and F-score

In addition to the EER/CER, there are also other metrics that try to reflect both types of errors, in order to give a more balanced indication of the overall performance of a binary classifier. The two most commonly used are **accuracy** and **F-score** (also called **F-measure**). Both metrics can be defined based on the four fundamental values (TP, TN, FP, and FN).

Accuracy of a binary classifier is defined as the fraction of *correctly predicted* samples (true positives and true negatives) among the total number of samples that have been classified, as shown in Equation 8.


(8) $$\begin{array}{l} \text{accuracy}=\frac{\text{TP}+\text{TN}}{\text{TP}+\text{TN}+\text{FP}+\text{FN}} \end{array}$$

The F-score of a binary classifier is actually a family of metrics. Its general form can be described based on a parameter β as defined in Equation 9.


(9) $$\begin{array}{l} F_{\beta }=\left(1+\beta^{2}\right)\cdot \frac{\text{precision}\cdot \text{recall}}{\beta^{2}\cdot \text{precision}+\text{recall}} \end{array}$$

The most widely used edition of all F-scores is the so-called **F1-score**, which is effectively the F-score with β = 1. More precisely, it is defined as shown in Equation 10.


(10) $$\begin{array}{l} F_{1}=2\cdot \frac{\text{precision}\cdot \text{recall}}{\text{precision}+\text{recall}}=\frac{2\text{TP}}{2\text{TP}+\text{FP}+\text{FN}} \end{array}$$

### 3.8 Receiver operating characteristic curve and area under curve

**Receiver operating characteristic** (ROC) curves are commonly used to measure the performance of binary classifiers that output a score (or probability) of prediction.

Consider the following. Let *S* be the set of all test samples and let the output scores *f*(*s*) (for all *s* ∈ *S*) lie in the interval [*a, b*] on the real line. Let *t* ∈ [*a, b*] be a prediction threshold for the model, and assume that the classifiers works as follows for all *s* ∈ *S*:


(11) $$\begin{array}{l} \text{class}\left(s\right)=\{\begin{array}{cc} \text{positive}, & \text{if}f\left(s\right)\geq t,\text{and}\\ \text{negative}, & \text{otherwise}. \end{array} \end{array}$$

Considering Equation 11, it is easy to see that, for *t* = *a*, all the samples will be classified as positive, leading to FN = TN = 0 so TPR = FPR = 1; while for *t* = *b*, all the samples will be classified as negative, leading to FP = TP = 0 so TPR = FPR = 0. For other threshold values between *a* and *b*, the values of TPR and FPR will normally be between 0 and 1. By changing *t* from *a* to *b* continuously, we can normally get a continuous curve that describes how the TPR and FPR values change from (0,0) to (1,1) on the 2D plane. This curve is the ROC curve of the binary classifier.

**Figure 1:**
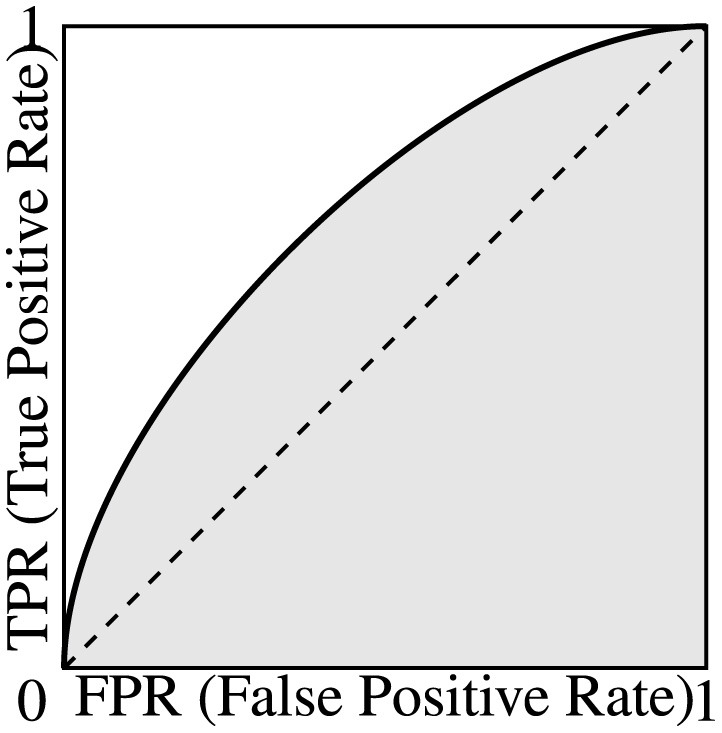
A representative ROC curve showing how TPR and FPR change w.r.t. the (hidden) threshold *t*. The area under the (ROC) curve (AUC) is shown in grey.

For a random classifier, assuming that *f*(*s*) distributes uniformly on [*a, b*] for the test set, we can mathematically derive its ROC curve being the TPR = FPR line, whose area under the ROC curve (AUC) is 0.5. For a binary classifier that performs better than a random predictor, we can also mathematically prove that its AUC is always higher than 0.5, with 1 being the best possible value. Note that no binary classifier can have an AUC below 0.5, since one can simply flip the prediction result to get a better predictor with an AUC of 1 − AUC. The relationship between the ROC and the AUC is graphically illustrated in Figure 1.

### 3.9 Log loss

Another widely used performance metric for binary classifiers that can return a probability score for the predicted label is **log loss**. For a binary classification with a true label *y* ∈ {0, 1} and an estimated probability *p* = Pr(*y* = 1), the log loss per sample is the negative log-likelihood of the classifier given the true label, defined as shown in Equation 12.


(12) $$\begin{array}{l} L_{\log}\left(y,p\right)=-\left(y\log\left(p\right)+\left(1-y\right)\log\left(1-p\right)\right) \end{array}$$

Given a testing set with *n* samples, the log loss score of a binary classifier can be calculated using Equation 13, where *y*_*i*_ is 1 if the *i*-th sample is true and 0 if false, and ŷ_*i*_ is the predicted probability of *y*_*i*_ = 1.


(13) $$\begin{array}{l} \text{LL}=-\frac{1}{n}\overset{n}{\underset{i=1}{\sum }}\left[y_{i}\log\left({ŷ}_{i}\right)+\left(1-y_{i}\right)\log\left(1-{ŷ}_{i}\right)\right] \end{array}$$

### 3.10 Extension to multi-class classifiers

All metrics that are defined based on the four basic values TP, TN, FP, and FN can be easily extended to **multi-class classification** by considering the prediction to be true or false individually with respect to each class. For example, if the system is classifying animals (cats, dogs, horses, lions, tigers, etc.), then a true positive prediction of an image to be of a cat, would simultaneously be true negative predictions for the remaining classes (dogs, horses, lions, tigers, etc.). If an image of a cat is incorrectly predicted to be that of a dog, it would be a false negative with respect to a cat, a false positive with respect to a dog, and a true negative with respect to all other classes.

### 3.11 Deception success rate

DSR aims to measure to what extent a deepfake detection model can be fooled by the generated deepfakes. It was used for the evaluation of the Audio Deep Synthesis Detection (ADD) 2022 Challenge (Yi et al., 2022a) and defined as follows:


(14) $$\begin{array}{l} \text{DSR}=\frac{W}{A*N} \end{array}$$

In Equation 14, W refers to the total number of incorrect detection samples by all the detection models under the condition of achieving each individual EER performance, A is the count of all the evaluation samples, and N is the number of detection models.

### 3.12 Perceptual quality assessment metrics

By definition, the main goal of deepfakes is to make it hard or impossible for human consumers (listeners or viewers) to distinguish fake media from real media. Therefore, when evaluating the quality of deepfake media, the quality perceived by human consumers of the media is key. This calls for a subjective assessment of the perceptual quality of the deepfake media as the “gold standard.” The most widely used subjective perceptual quality assessment (PQA) metric for audio-visual signals is **mean opinion score** (MOS), which has been widely used by the signal processing and multimedia communication communities, including digital TV and other multimedia-related consumer applications. As its name implies, MOS is calculated by averaging the subjective scores given by a number of human judges, normally following a numerical scale between 1 and 5 or between 0 and 100. MOS has been used in some deepfake-related challenges and also for evaluating and comparing the quality (realness/naturalness) of deepfake datasets (see Section 4.7).

As a general subjective PQA metric, MOS has been standardised by the ITU.^6^ There are also ITU standards defining more specific subjective Video Quality Assessment (VQA) metrics and the standard procedures one should follow to conduct VQA user studies, e.g., ITU-T Recommendation P.910 “Subjective video quality assessment methods for multimedia applications.”^7^ Note that the ITU standards focus more on traditional perceptual quality, i.e., how good a signal looks or sounds, even if it looks or sounds not real (e.g., too smooth). On the other hand, for deepfakes, the focus is rather different because what matters is the realness and naturalness of the created media, i.e., how real and natural it looks or sounds, even if it is of low quality. To some extent, we can also consider realness and naturalness as a special aspect of perceptual quality.

One major problem of subjective PQA metrics like MOS is the need to recruit human judges and to have a well-controlled physical testing environment and protocol, which are not easy for many applications. To help reduce the efforts and costs of conducting PQA-related user studies, various objective PQA metrics have been proposed, where the term “objective” refers to the fact that such metrics are human-free, i.e., automatically calculated following a computational algorithm or process. Depending on whether a reference exists, such objective PQA metrics can be largely split into three categories: full-reference (FR) metrics (when the original “perfect-quality” signal is available as the reference), reduced-reference (RR) metrics (when some features of the original “perfect-quality” signal are available as the reference), and no-reference (NR) metrics (when the original signal is unavailable or such an original signal does not exist). For deepfakes, normally NR or RR metrics are more meaningful because the “fake” part of the word means that part of the whole data does not exist in the real world, hence a full reference cannot be obtained. RR metrics are still relevant because deepfakes are often produced for a target's specific attributes (e.g., face and voice), where the reduced reference will be such attributes. NR metrics will be useful to estimate the realness and naturalness of a deepfake, simulating how a human judge would rate it in a controlled subjective PQA user study.

PQA is a very active research area and many PQA metrics have been proposed, some of which have been widely used in real-world products and services, e.g., **mean squared error** (MSE), **peak signal-to-noise ratio** (PSNR), and **structural similarity index measure** (SSIM) for FR PQA of digital images and videos defined as in Equations 15–17, respectively, where $X={\left\{x_{i}\right\}}_{i}^{n}$ is the reference (the original signal), $Y={\left\{y_{i}\right\}}_{i}^{n}$ is the signal whose visual quality is assessed, *n* is the number of pixels in *X* and *Y*, *L* is the maximum possible pixel value of *X* and *Y* (e.g., 255 for 8-bit grey-scale images), $c_{1}={\left(k_{1}L\right)}^{2}$ and $c_{2}={\left(k_{2}L\right)}^{2})$ are two stabilising parameters (*k*_1_ = 0.01 and *k*_2_ = 0.03 by default). For more about PQA metrics for different types of multimedia signals, we refer readers to some relevant surveys (Akhtar and Falk, 2017; Pal and Triyason, 2018; Zhai and Min, 2020).


(15) $$\begin{array}{l} \text{MSE}\left(X,Y\right)=\overset{n}{\underset{i=1}{\sum }}\left(y_{i}-x_{i}\right) \end{array}$$

(16) $$\begin{array}{l} \text{PSNR}\left(X,Y\right)=10\log_{10}\left(\frac{L^{2}}{\text{MSE}}\right) \end{array}$$

(17) $$\begin{array}{l} \text{SSIM}\left(X,Y\right)=\frac{\left(2\mu_{x}\mu_{y}+c_{1}\right)\left(2\sigma_{xy}+c_{2}\right)}{\left(\mu_{x}^{2}+\mu_{y}^{2}+c_{1}\right)\left(\sigma_{x}^{2}+\sigma_{y}^{2}+c_{2}\right)} \end{array}$$

### 3.13 Multimodal alignment assessment metrics

With the increasing amount of research on leveraging multimodalities in deepfake generation (e.g., text-to-image, text-to-video, and audio-to-video), new evaluation metrics became needed to assess to what extent the covered modalities are aligned. The development of such metrics is challenging as they require a comprehensive and fine-grained cross-modal understanding (Huang et al., 2023).

While MAA metrics have been used to assess different tasks (e.g., image-caption alignment), they can also be considered an alternative to PQA metrics for deepfake evaluation. For instance, with the introduction of state-of-the-art text-to-image models, such as DALL-E,^8^ which enables easier generation of deepfake images from natural language descriptions, text-to-image alignment metrics can be utilised to assess whether the generated image aligns the provided description. With this respect, several metrics based on vision-language models, which can simultaneously learn from images/videos and texts [e.g., BLIP (Li J. et al., 2022) and CLIP (Radford et al., 2021)], have been proposed. Some examples of such metrics include **ClipScore** (Hessel et al., 2021), **disentangled BLIP-VQA** (Huang et al., 2023), and **BLIP-CLIP** (Chefer et al., 2023) for text-to-image generation, as well as **X-CLIP** (Ni et al., 2022) for text-to-video generation.

In addition to vision-language models, MAA metrics have also made use of multimodal large language models by generating a *chain-of-thought* (Wei et al., 2022) through prompts. For example, **X-IQE** (Chen et al., 2023) leverages MiniGPT-4 (Zhu et al., 2024) with prompts prepared with the help of art professionals to evaluate the fidelity, alignment, and aesthetics of the generated images. Another example is **T2VScore-A** (Wu et al., 2024), which uses GPT-3.5 to identify questions, choices, and answers to evaluate alignment between a generated video and a given text prompt. It is then measured based on the accuracy of visual question answering.

While existing MAA metrics mostly focus on the semantic alignment of modalities, temporal alignment is also worth considering for the MAA task. With this respect, **AV-Align** (Yariv et al., 2024) is a metric for assessing temporal alignment between audio-video pairs. It is based on energy peaks of both modalities, i.e., the highest mean magnitude of optical flow for video frames and the onset of the audio waveform.

### 3.14 More about standards

Many of the basic performance metrics described in this section have been widely used by deepfake researchers as de facto standards, e.g., EER, log loss and MOS have been widely used in deepfake-related challenges. Also, the combination of precision, recall and F1-score has been widely used to assess the performance of binary classifiers. While there have been a number of ITU standards on PQA to date, there do not seem to be many standardisation efforts on the performance metrics for the evaluation of binary classifiers. This was the case until at least 2017 when ISO and IEC jointly set up the ISO/IEC JTC 1/SC 42,^9^ a standardisation subcommittee (SC) focusing on AI under ISO/IEC JTC 1,^10^ the joint technical committee for standardising “information technology.”

One recent effort that ISO/IEC JTC 1/SC 42 made is to produce the ISO/IEC TR 24029-1:2021 “Artificial Intelligence (AI)—Assessment of the robustness of neural networks—Part 1: Overview,”^11^ a technical report (TR) that systematically covers many commonly used performance assessment concepts, methods, and metrics. Although the technical report has “neural networks” in its title, most performance assessment concepts, methods, and metrics included are common ones for all supervised machine learning models.

In terms of performance metrics, two other ongoing work items of the ISO/IEC JTC 1/SC 42 that deserve attention are as follows:

- ISO/IEC DTS (Draft Technical Specification) 4,213 “Information technology—Artificial Intelligence— Assessment of machine learning classification performance”^12^
- ISO/IEC AWI (Approved Work Item) TS (Technical Specifications) 5,471 “Artificial intelligence—Quality evaluation guidelines for AI systems”^13^

While the ISO/IEC JTC 1/SC 42 was created very recently, another standardisation subcommittee under ISO/IEC JTC1 has a much longer history of nearly 20 years: the ISO/IEC JTC 1/SC 37^14^ that focuses on biometrics-related technology. This standardisation subcommittee is highly relevant for deepfake since deepfake faces can be used to spoof biometrics-based user authentication systems. In this context, the following three standards are of particular relevance:

**ISO/IEC 19795-1:2021 “Information technology—Biometric performance testing and reporting—Part 1: Principles and framework:”**^15^ This standard covers general metrics about evaluating biometric systems. Two major metrics in this context are **false accept rate** (FAR) and **false reject rate** (FRR), which refer to the standard FPR and FNR, respectively. This standard also deprecates the use of single-number metrics including the EER and AUC (which were widely used in biometrics-related research in the past).

**ISO/IEC 30107-1:2016 “Information technology—Biometric presentation attack detection—Part 1: Framework:”**^16^ This standard defines a general framework about **presentation attack detection** (PAD) mechanisms, where the term “**presentation attack**” refers to the “*presentation of an artefact or of human characteristics to a biometric capture subsystem in a fashion intended to interfere with system policy*.” It focuses on biometric recognition systems, where a PAD mechanism is a binary classifier trying to predict presentation attacks (also called attack presentations, e.g., fake faces) as positive and bona fide (real) presentations as negative.

**ISO/IEC 30107-3:2017 “Information technology—Biometric presentation attack detection—Part 3: Testing and reporting:”**^17^ This standard defines a number of special performance metrics for evaluating PAD mechanisms standardised in the ISO/IEC 30107-1:2016. Three such metrics look at error rates: **attack presentation classification error rate** (APCER) referring to the standard FPR, **normal/bona fide presentation classification error rate** (NPCER/BPCER) referring to the standard FNR, and **average classification error rate** (ACER) that is defined as the average of the APCER and the NPCER/BPCER. Such metrics have been used in biometrics-related challenges such as Face Anti-spoofing (Presentation Attack Detection) Challenges.^18^ When deepfake images or videos are used to spoof a biometric system, such standardised metrics will become relevant.

### 3.15 Discussion: performance metrics and standards

This section provides a comprehensive summary of performance metrics used for evaluating and benchmarking deepfake generators and detectors. It is rare that all such metrics are used for a specific application. Instead, one or several are chosen based on specific needs. For a deepfake detection system as a binary classifier, many researchers have chosen to use overall metrics such as accuracy, AUC, EER and log loss, but the combination of precision, recall and F1-score is also common. However, there is a growing interest in using more deepfake-focused metrics, including PQA and MAA metrics, especially for evaluating deepfake generation. Other than general evaluation metrics, some deepfake-related challenges and competitions have introduced their own specific metrics, such as DSR. Furthermore, there exist several metrics specific to certain deepfake-related tasks, including code generation, animation generation, and synthetic data generation (Bandi et al., 2023). The use of different performance metrics can make the comparison of different reported results more difficult, so we hope the expected new ISO/IEC standard particularly ISO/IEC 4213 will help.

It is worth mentioning that, in addition to evaluating the performance of deepfake detectors, the introduced performance metrics for evaluating binary classifiers can also be used to evaluate the performance of deepfake generation methods by considering how deepfake detectors fail. For instance, organisers of the Voice Conversion Challenge 2018 and 2020 used this approach to benchmark how well voice conversion (VC) systems can generate high-quality fake speech samples.

Another point we would like to mention is that for deepfake videos there are two levels of performance metrics: those at the frame level (metrics of each frame), and those at the video level (metrics for the whole video). Generally speaking, the latter can be obtained by averaging the former for all frames, potentially following an adaptive weighting scheme, so that more important (key) frames will be counted more.

## 4 Deepfake-related datasets

In this section, we cover all deepfake-related datasets we identified from the meta-review of deepfake-related survey papers, deepfake-related challenges, three online collections of deepfake-related datasets on GitHub,^19^^–^^21^ and the co-authors' personal collections. We explain the datasets covered in five categories: deepfake image datasets, deepfake video datasets, deepfake audio/speech datasets, deepfake text datasets, and hybrid deepfake datasets (mainly mixed image and video datasets).

Note that many datasets of real (authentic) media were also used by deepfake researchers for two purposes. First, any detectors would need both fake and real media to demonstrate their performance. Second, real media have also been used to train deepfake generators as the training set. In this section, we include only datasets containing deepfake media, some of which contain both deepfake and real media.

Some datasets, especially those created for deepfake-related challenges and competitions, have separate subsets for training and evaluation (testing) purposes. The split is necessary for such challenges and competitions, but not very useful for people who just want to use such datasets. Therefore, in this section when introducing such datasets we will ignore that level of details and focus on the total number of data including the number of real and fake samples.

### 4.1 Deepfake image datasets

Table 2 shows basic information about the image datasets covered.

**Table 2:** Deepfake-related image datasets.

**Dataset**	**Size**	**Year**	**Generation method**
SwapMe & FaceSwap	4,310 images	2017	Face swapping
Fake Faces in the Wild (FFW)	53,000 images	2018	Fake YouTube videos
generated.photos datasets	2.7 million images	Since 2018	StyleGAN
MesoNet Deepfake Dataset	19,509 images	2018	Face extraction from forged videos
100K-Generated-Images	100,000 images	2019	A GAN generator
Ding et al.'s swapped face dataset	420,053 images	2019	Face swapping
iFakeFaceDB	87,000 images	2019	StyleGAN + GANprintR
Faces-HQ	40,000 images	2019-20	Collection from other datasets
CelebA-Spoof	625,537 images	2020	Face spoofing
Diverse Fake Face Dataset (DFFD)	299,039 images	2020	Multiple facial manipulation methods
DiffusionForensics	615,200 images	2023	Pretrained diffusion models
DeepFakeFace (DFF)	120,000 images	2023	Diffusion models + Face manipulation methods

**SwapMe and FaceSwap dataset** (Zhou et al., 2017): This dataset contains 4,310 images, including 2,300 real images and 2,010 fake images created using FaceSwap^22^ and the SwapMe iOS app (now discontinued). The fake images were generated by tampering with one face in each authentic image with face swapping. The selected images cover diverse events, genders, ages, and races.

**Fake Faces in the Wild (FFW) dataset** (Khodabakhsh et al., 2018): This dataset contains 131,500 face images, including 78,500 bona fide images extracted from 150 videos in the FaceForensics dataset and 53,000 fake images extracted from 150 fake videos collected from YouTube. The fake images involve both tampered images and those generated using CGI.

**generated.photos datasets**^23^: This is a number of commercial datasets provided by Generated Media, Inc., with up to nearly 2.7 million synthetic face images generated by StyleGAN. A free edition with 10,000 128x128 synthetic images is made available for academic research. The website also provides an interactive face generator^24^ and an API.^25^ The generated.photos datasets have a good diversity: five age groups (infants, children, youth, adults, middle-aged), two genders (male and female), four ethnicities (white, black, Latino, Asian), four eye colours (brown, grey, blue, green), four hair colours (brown, black, blond, grey), three hair length (short, medium, long), facial expressions, three head poses (front facing, left facing, right facing), two emotions (joy and neutral), two face styles (natural, beautified).^26^

**MesoNet Deepfake dataset** (Afchar et al., 2018): This dataset includes 19,457 face images,^27^ including 7,948 deepfake images generated from 175 forged videos collected online and 11,509 real face images collected from various online sources. The face images were extracted from the collected videos by utilising a visual object detection method, and around 50 faces per scene were obtained.

**100K-Generated-Images** (Karras et al., 2019): This dataset includes 100,000 synthesised face, bedroom, car and cat images by a GAN generator trained based on real images in the FFHQ^28^ and LSUN^29^ datasets (three object types—bedrooms, cars and cats—for the latter). Note that the name “100K-Generated-Images” was not a proper one as the authors (Karras et al., 2019) just used this to name a sub-folder of their Google Drive shared space, but it was used in one of the survey papers (Tong et al., 2020).

**Ding et al. (****2020****)'s swapped face dataset**: This dataset contains 420,053 images of celebrities, including 156,930 real ones downloaded using Google Image API and 263,123 fake face-swapped ones created using two different methods (Nirkin's method and Auto-Encoder-GAN). While the former method consisted of multiple techniques pipelined, the latter was fully automated based on a CNN architecture.

**iFakeFaceDB** (Neves et al., 2020): This dataset includes 87,000 224 × 224 face images, generated by processing some StyleGAN-generated synthetic images using the GAN-fingerprint Removal approach (GANprintR) proposed by Neves et al. (2020). It is the replaced version of the **FSRemovalDB** dataset, which contains 150,000 face images generated using an earlier version of GANprintR.

**Faces-HQ** (Durall et al., 2019): This dataset includes 40,000 high-resolution images, half real and half deepfake. The images were collected from four sources: the CelebA-HQ dataset,^30^ the Flickr-Faces-HQ dataset (see text footnote ^28^), the 100K-Faces dataset^31^ (not available any longer, see the description of generated.photos datasets), and thispersondoesnotexist.com.

**CelebA-Spoof** (Zhang Y. et al., 2020): This dataset includes 625,537 synthesised face images of 10,177 celebrities, with 43 rich attributes on face, illumination, environment and spoof types. The real images were selected from the CelebA dataset.^32^ The 43 attributes include 40 for real images, covering all facial components and accessories (e.g., skin, nose, eyes, eyebrows, lip, hair, hat, and eyeglass), and 3 for fake images, covering spoof types, environments and illumination conditions.

**Diverse fake face dataset** (Dang et al., 2020): This dataset contains 299,039 images, including 58,703 real images sampled from three datasets [FFHQ (see text footnote ^28^), CelebA (see text footnote ^32^), and FaceForensics++^33^] and 240,336 fake ones in four main facial manipulation types (identity swap, expression swap, attribute manipulation, and entire synthesis). The images cover two genders (male and female), a wide age group (the majority between 21 and 50 years old), and both low- and high-quality levels.

**DiffusionForensics** (Wang et al., 2023a,b): This dataset contains 615,200 diffusion-generated images, sourced from LSUN-Bedroom (see text footnote ^29^), ImageNet (Deng et al., 2009), and CelebA-HQ (see text footnote ^30^) datasets. The dataset covers images that belong to one of the following image generation methods—unconditional, conditional, and text-to-image generation, and contains three fields—source image, reconstructed image, and the image referring to the error between these images. Moreover, 11 different generators were leveraged for the generation of the fake images.

**DeepFakeFace (DFF)** (Song et al., 2023a,b): This dataset contains 30,000 real and 90,000 fake celebrity images. The real images were retrieved from the IMDB-Wiki (Rothe et al., 2015) dataset, and the fake ones were generated by utilising three different methods—Stable Diffusion v1.5 and Stable Diffusion Inpainting diffusion models, as well as the InsightFace toolbox containing several face manipulation algorithms.

### 4.2 Deepfake video datasets

Table 3 shows basic information about the video datasets covered.

**Table 3:** Deepfake-related video datasets.

**Dataset**	**Size**	**Year**	**Language/ethnicity**	**Generation method**
DeepfakeTIMIT	620 videos	2018	English	GAN-based face swapping
FaceForensics	1,004 videos	2018	Not specified	Face manipulation
UADFV	98 videos	2018	Not specified	Multiple GANs
FaceForensics++	5,000 videos	2019	Not specified	Multiple face manipulation methods
Deep Fakes Dataset	142 videos	2020	Not specified	Collection
Celeb-DF v1	1,203 videos	2020	Multiple ethnic groups	Face swapping
Celeb-DF v2	6,229 videos	2020	Multiple ethnic groups	Face swapping
DFD	3,363 videos	2019	Not specified	Face swapping
DeeperForensics-1.0	60,000 videos	2020	26 nationalities	Face swapping
DFDC (full)	128,154 videos	2020	Not specified	Face/Audio swapping + GANs
WildDeepfake	7,314 face sequences	2020	Not specified	Online collection
FFIW10*K*	20,000 videos	2021	Multilingual	Face swapping
KoDF	37,942 videos	2021	Korean subjects	Face swapping + Face reenactment
VideoForensicsHQ	1,737 videos	2021	Not specified	Deep Video Portraits (DVP)
DF-W	1,869 videos	2021	Not specified	Online collection
FMFCC-V	82,392 videos	2022	Asian subjects	Face swapping
DF-Platter	133,260 videos	2022	Indian subjects	Face swapping
AV-Deepfake1M	1,146,760 videos	2023	Not specified	Face reenactment + Text-to-speech
DFDM	6,450 videos	2022	Not specified	Face swapping

**DeepfakeTIMIT** (Korshunov and Marcel, 2019): This dataset contains 620 deepfake face videos, generated by GAN-based face swapping without manipulation of audio, covering 32 subjects and two quality levels (high and low). The videos in the dataset are recordings of people facing the camera and reciting predetermined short English sentences.

**FaceForensics (FF)** (Rössler et al., 2018): This dataset contains 1,004 face videos with over 500,000 frames, covering various quality levels and two types of facial manipulation using the Face2Face approach. This dataset is now replaced by the larger FaceForensics++ dataset (see below).

**FaceForensics++ (FF++)** (Rössler et al., 2019): This dataset contains 5,000 face videos with over 1.8 million manipulated frames, including 1,000 real videos (with 509,914 frames) downloaded from YouTube, and 4,000 fake videos created using four face manipulation methods (Deepfakes, Face2Face, FaceSwap and NeuralTextures). The videos cover two genders (male and female), and three quality levels (VGA/480p, HD/720p, and FHD/1080p).

**UADFV dataset** (Li et al., 2018): This dataset contains 98 face videos, half (49) are real ones downloaded from YouTube, and the other half are fake ones generated using the FakeApp mobile application (which is now discontinued). The video dataset was created to demonstrate a deepfake video detection method based on the detection of eye-blinking behaviours, so all the videos contain at least one eye-blinking event. All fake videos were created by swapping the original face in each of the real videos with the face of the actor Nicolas Cage,^34^ thus, only one subject is represented.

**Deep fakes dataset** (Ciftci et al., 2020): This dataset contains 142 “in the wild” deepfake portrait videos, collected from a range of online sources including news articles, online forums, mobile apps, and research presentations. The videos are diverse, covering the source generative model, resolution, compression, illumination, aspect ratio, frame rate, motion, pose, cosmetics, occlusion, content, and context.

**Celeb-DF v1**^35^: This dataset contains 1,203 face videos of celebrities, including 408 real videos collected from YouTube with subjects of different ages, ethnic groups and genders, and 795 deepfake videos synthesised from these real videos.

**Celeb-DF v2** (Li Y. et al., 2020): This dataset contains 6,229 face videos of celebrities, including 590 real videos collected from YouTube with subjects of different ages, ethnic groups and genders, and 5,639 deepfake videos synthesised from these real videos. The deepfake videos were generated by swapping faces for each pair of the 59 celebrities, using an improved DeepFake synthesis algorithm.

**DeepFake detection dataset** (Dufour and Gully, 2019): This dataset contains 3,363 face videos, covering 28 subjects, gender, and skin colour. It was created as a joint effort between two units of Google, Inc.: Google AI^36^ and JigSaw.^37^ The deepfake videos were generated by using publicly available face-swapping methods although no more details were provided.

**DeeperForensics-1.0** (Jiang et al., 2020): This dataset contains 60,000 indoor face videos (with 17.6 million frames) generated by face swapping, covering 100 subjects, four skin tones (white, black, yellow, and brown), two genders (male and female), different age groups (20–45), 26 nationalities, 7 different angles, 8 face expressions, and different head poses.

**DFDC (Deepfake Detection Challenge) full dataset** (Dolhansky et al., 2020): This dataset contains 128,154 face videos of 960 subjects, including 23,654 real videos from 3,426 paid actors and 104,500 deepfake videos created using eight different methods (DF-128, DF-256, MM/NN face swap, NTH, FSGAN, StyleGAN, refinement, and audio swap).

**WildDeepfake** (Zi et al., 2020): This dataset contains 7,314 face sequences extracted from 707 deepfake videos that were collected completely from the Internet. It covers diverse scenes, multiple persons in each scene and rich facial expressions. Different from other deepfake video datasets, WildDeepfake contains only face sequences, not the full videos. This makes the dataset more like between an image dataset and a video one. We decided to keep it in the video category since the selection process was still more video-focused.

FFIW10*K* **(Face Forensics in the Wild) dataset** (Zhou et al., 2021): This dataset contains 10,000 real and 10,000 high-quality forgery videos, with video- and face-level annotations. The dataset focuses on a more challenging case for forgery detection: each video involves one to 15 individuals, but only some (a minority of) faces are manipulated. The deepfake videos were generated by utilising three different face-swapping methods—two learning-based methods, DeepFaceLab and FSGAN, as well as a graphic-based method, FaceSwap.

**Korean DeepFake Detection Dataset** (Kwon et al., 2021): This dataset contains 37,942 videos of paid subjects (395 Koreans and 8 Southeastern Asians), including 62,166 real videos and 175,776 fake ones created using six methods, including three face-swapping methods (i.e., FaceSwap, DeepFaceLab, and FSGAN), one video-driven face reenactment method [i.e., First Order Motion Model (FOMM)], and two audio-driven face reenactment method [i.e., Audio-driven Talking Face HeadPose (ATFHP) and Wav2Lip]. The videos cover a balanced gender ratio and a wide range of age groups.

**VideoForensicsHQ** (Fox et al., 2021): This dataset contains 1,737 videos with 1,666,816 frames, including 1,339,843 real frames and 326,973 fake frames generated using Deep Video Portraits (DVP) (Kim et al., 2018), i.e., a method that enables to transfer head pose, facial expression, and eye motion of the source while preserving the target's identity and appearance. The original videos were obtained from three sources: the dataset used in (Kim et al., 2019), the Ryerson Audio-Visual Database of Emotional Speech and Song (RAVDESS) (Livingstone and Russo, 2018), and YouTube. Most videos have a resolution of 1280 × 720.

**DF-W** (Pu et al., 2021a,b): This dataset contains 1,869 real-world deepfake videos collected from two online video portals: YouTube (1,062) and Bilibili^38^ (807). The authors also collected the same number of real videos from 6 research community datasets for the results reported in their paper (Pu et al., 2021a), but they chose not to release such videos as part of DF-W.

**FMFCC-V** (Li G. et al., 2022a,b): This dataset contains 38,102 deepfake videos and 44,290 pristine videos, corresponding to over 23 million frames. It was created by a group of Chinese researchers from the State Key Laboratory of Information Security, Institute of Information Engineering, Chinese Academy of Sciences, for the accompanying video track of the first Fake Media Forensics Challenge of the China Society of Image and Graphics. The source videos were collected from 83 paid Asian (likely all Chinese) individuals. Then, the synthesised videos were generated by leveraging four face-swapping methods—Faceswap, Faceswap-GAN, DeepFaceLab, and Recycle-GAN.

**DF-Platter** (Narayan et al., 2023a,b): This dataset contains 764 real videos of 454 Indian individuals, and 133,260 deepfake videos generated using three state-of-the-art synthesis methods: FSGAN (Nirkin et al., 2019), FaceSwap, and FaceShifter (Li L. et al., 2020). The videos were collected in the wild, particularly from YouTube, considering many diversity factors, such as gender, orientation, skin tone, face size (counted in pixels), lighting conditions, background, and in the presence of occlusion. Each video lasts approximately 20 seconds in duration.

**AV-Deepfake1M** (Cai et al., 2023a,b): This dataset contains over 1 million videos (286,721 real and 860,039 fake), corresponding to 1,886 hours of audio-visual data, generated from 2,068 unique subjects. The dataset covers different video manipulation techniques, including fake audio over fake visuals, fake audio over real visuals, and real audio over fake visuals. The fake audios were generated by an identity-dependent text-to-speech method, VITS, while the TalkLip model was used for face reenactment to generate lip-synchronised fake visual frames.

**Deepfakes from different models** (Jia et al., 2022a,b): This dataset contains 6,450 face-swap deepfake videos generated by five different Autoencoder models based on the Faceswap^39^ software (i.e., Faceswap, Lightweight, IAE, Dfaker, and DFL-H128). For the generation of deepfake videos, real videos in the Celeb-DF dataset were used. The dataset is available upon request.

### 4.3 Deepfake audio/speech datasets

Table 4 shows basic information about the audio/speech datasets covered.

**Table 4:** Deepfake-related audio datasets (**VC**: Voice Conversion, **TTS**: Text-to-speech, **AS**: Audio Splicing, **SA**: Spoofing Attack, **VS**: Voice Synthesis, **SE**: Speech Enhancement, **CMF**: Copy-Move Forgery).

**Dataset**	**Size**	**Year**	**Language**	**Generation method**
VCC 2016 dataset	3,078 utterances	2016	English	VC
VCC 2018 dataset	2,582 utterances	2018	English	VC
VCC 2020 dataset	3,505 utterances	2020	English, Finnish, German, Chinese	VC
ASVspoof 2019 dataset (LA task)	121,461 utterances	2019	English	VS, VC
ASVspoof 2021 dataset (LA task)	164,640 utterances	2021	English	VC, TTS, Hybrid SA
ASVspoof 2021 dataset (DF task)	593,253 utterances	2021	English	SA
ReMASC	54,712 recordings	2019	English	VS
FSD	650 songs	2023	Chinese	VC, VS
DECRO	118,382 utterances	2023	English, Chinese	SA
WaveFake	134,268 audios	2021	English, Japanese	TTS
HAD	160,836 audios	2021	Chinese	TTS, AS
ADD 2022 dataset	154,949 audios	2022	Chinese	VS, VC
CMFD	5,600 audios	2022	English, Chinese	CMF
In-the-Wild	31,779 audios	2022	English	Online collection
SceneFake	84,480 audios	2022	English	SE
EmoFake	88,200 audios	2022	English	VC
PartialSpoof	121,461 audios	2021–2022	English	VC, TTS, AS
CFAD	331,600 audios	2023	Chinese	TTS, AS
ADD 2023 dataset	517,068 utterances	2023	Chinese	VC, TTS, AS
MLAAD	163.9 hours of synthetic voice	2024	23 languages	TTS

Voice conversion (VC) is a technology that can be used to modify an audio and speech sample so that it appears as if spoken by a different (target) person than the original (source) speaker. Obviously, it can be used to generate deepfake audio/speech samples. The biennial Voice Conversion Challenge^40^ that started in 2016 is a major challenge series on VC. Datasets released from this challenge series are very different from other deepfake datasets: the deepfake data is not included in the original dataset created by the organisers of each challenge, but in the participant submissions (which are retargeted/fake utterances produced by VC systems built by participants). The challenge datasets also include the evaluation (listening-based) results of all submissions. Some fake utterances may be produced by DL-based VC systems, so we consider all datasets from this challenge series relevant for the purpose of this survey.

**Voice conversion challenge 2016 database** (Toda et al., 2016): The original dataset created by the challenge organisers was derived from the DAPS (Device and Produced Speech) Dataset (Mysore, 2015). It contains 216 utterances (162 for training and 54 for testing) per speaker from 10 speakers. Participating teams (17) developed their own VC systems for all 25 source-target speaker pairs and then submitted generated utterances for evaluation. At least six participating teams used DL-related techniques (LSTM, DNN) in their VC systems (see Table 2 of the result analysis paper^41^), so the submitted utterances can certainly be considered deepfakes.

**Voice conversion challenge 2018 database** (Lorenzo-Trueba et al., 2018): The original dataset created by the challenge organisers was also based on the DAPS dataset. It contains 116 utterances (81 for training and 35 for testing) per speaker from 12 speakers in two different tasks (called Hub and Spoke). Participating teams (23 in total, all for Hub and 11 for Spoke) developed their own VC systems for all 16 source-target speaker pairs and then submitted generated utterances for evaluation. Compared to the 2016 challenge, more participating teams used DL-related techniques (e.g., WaveNet, LSTM, DNN, CycleGAN, DRM – deep relational models, and ARBM – adaptive restricted Boltzmann machines) in their VC systems.

**Voice conversion challenge 2020 database** (Yi et al., 2020): This dataset is based on the Effective Multilingual Interaction in Mobile Environments (EMIME) dataset,^42^ a bilingual (Finnish/English, German/English, and Mandarin/English) database. It contains 145 utterances (120 for training and 25 for testing) per speaker from 14 speakers for two different tasks (with 4 × 4 and 4 × 6 source-target speaker pairs, respectively). Participating teams (33 in total, out of which 31 for Task 1 and 28 for Task 2) developed their own VC systems for all source-target speaker pairs and then submitted generated utterances for evaluation. Compared to the 2018 challenge, DL-based VC systems were overwhelmingly used by almost all participating teams (i.e., WaveNet and WaveGAN were among the most used DL-based building blocks).

A major set of deepfake speech datasets were created for the **ASVspoof** (Automatic Speaker Verification Spoofing and Countermeasures) Challenge^43^ (2015–2021, held biannually). The datasets for the 2019 and 2021 challenges contain speech data that can be considered deepfakes.

**ASVspoof 2019 challenge database** (Wang et al., 2020): This dataset is based on the Voice Cloning Toolkit (VCTK) corpus,^44^ a multi-speaker English speech database captured from 107 speakers (46 males and 61 females). Two attack scenarios were considered: logical access (LA) involving spoofed (synthetic or converted) speech, and physical access (PA) involving replay attacks of previously recorded bona fide recordings). For our purpose in this survey, the LA scenario is more relevant. The LA part of the dataset includes 12,483 bona fide (real) utterances and 108,978 spoofed utterances. Some of the spoofed speech data for the LA scenario were produced using a generative model involving DL-based techniques such as long short-term memory (LSTM),^45^ WaveNet (Oord et al., 2016), WaveRNN (Kalchbrenner et al., 2018), and WaveCycleGAN2 (Tanaka et al., 2019). Note that the challenge organisers did not use the term “deepfake” explicitly, despite the fact that the DL-generated spoofed speech data can be considered as deepfakes.

**ASVspoof 2021 challenge—logical access database** (Delgado et al., 2021a; Liu et al., 2023): This dataset contains 16,492 bona fide and 148,148 spoofed speech data for the logical access (LA) task. The bona fide speech data in the dataset were sourced from the ASVspoof 2019 LA evaluation database, and the spoofed data were generated by leveraging 13 voice conversion, text-to-speech, and hybrid spoofing attack algorithms.

**ASVspoof 2021 challenge—speech deepfake database** (Delgado et al., 2021b; Liu et al., 2023): In 2021, the challenge included an explicitly defined track on deepfake, but the task description suggests that the organisers of the challenge considered a broader definition of the term “deepfake” by looking at spoofing human listeners rather than ASV (Automatic Speaker Verification) systems. The dataset includes 20,637 bona fide and 572,616 spoofed speech data. Other than the ASVspoof 2019 LA evaluation database, the bona fide speech data in this dataset were also sourced from VCC 2018 and VCC 2020 datasets. When it comes to spoofed data generation, over 100 different spoofing attack algorithms were used.

**ReMASC** (Gong et al., 2019a,b): This dataset contains 9,240 genuine and 45,472 replayed recordings of voice commands in English performed by English, Chinese, and Indian native speakers. Although the dataset mainly focuses on replay attacks rather than synthesised speech, it also contains fake videos generated using two speech synthesis methods. The dataset also includes a quick evaluation set, which is a small but representative dataset with around 2,000 samples, that can be used for the quick evaluation of developed audio deepfake detection models.

**Fake song detection dataset** (Xie et al., 2023a,b): This dataset contains 200 real and 450 fake songs in Chinese. To generate fake songs, initially, fake singing voices were generated by five state-of-the-art singing voice synthesis (DiffSinger) and singing voice conversion methods (RVC and three variations of SO-VITS). Then, instrumental tracks were extracted from real songs and combined with fake singing voices.

**DEepfake CROss-lingual dataset** (Ba et al., 2023a,b): This dataset consists of English and Chinese speech samples. The English part of the dataset contains 12,484 real and 42,800 fake utterances while the Chinese part consists of 21,218 real and 41,880 fake utterances. For the real speech samples, six Chinese recording datasets and the ASVspoof2019 LA (Wang et al., 2020) dataset were used. The fake utterances, however, were generated by leveraging 10 different spoofing attack algorithms, including HiFiGAN, Multiband-MelGAN, PWG, Tacotron, FastSpeech2, StarGANv2, VITS, NVCNet, Baidu, and Xunfei.

**WaveFake** (Frank and Schönherr, 2021a,b): This dataset contains 16,283 real audio clips and 117,985 fake audio clips in English and Japanese, which corresponds to 175 h of audio data. The reference datasets for real videos include LJSPEECH for English and JSUT for Japanese. For the generation of fake audio clips, six different GAN-based architectures (i.e., MelGAN, PWG, Multi-band MelGAN, Full-band MelGAN, HiFi-GAN, and WaveGlow) were leveraged.

**Half-truth audio detection dataset** (Yi et al., 2021): This dataset focuses on the attack scenarios in which the attacker hides small fake audio clips into real speech audio. The dataset comprises 53,612 real audio clips and 107,224 fake audio clips in Chinese. The fake audio clips in the dataset cover both fully fake and partially fake samples. While the fully fake samples were generated utilising a text-to-speech method, namely GST Tacotron,^46^ the latter set of videos were based on the manipulation of the audio segments corresponding to selected keywords in the original utterances.

**ADD 2022 dataset** (Yi et al., 2022a): This is the original dataset of the first audio deep synthesis detection challenge, held in 2022. The dataset covers data for the three tracks of the challenge—low-quality fake audio detection (LF), partially fake audio detection (PF), and audio fake game (FG). In total, the dataset comprises 16,257 real audio samples and 138,692 fake audio samples in Chinese. The dataset is based on common Mandarin speech datasets, including AISHELL-1, AISHELL-3, and AISHELL-4, and the fake audios were generated using mainstream speech synthesis and voice conversion methods.

**Copy-Move forged dataset** (Su et al., 2023a,b): This dataset contains 3,600 real audio samples and 2,000 fake audio samples in two languages – English and Chinese. The dataset was based on Librispeech and Chinspeech speech datasets, and the fake samples were generated by applying copy-move forgery to the samples of these two datasets.

**In-the-Wild** (Müller et al., 2022): This dataset focuses on detecting audio deepfakes for politicians and celebrities. It comprises 19,963 real speeches and 11,816 fake audio samples of 58 public figures, derived from publicly available sources. In total, the dataset corresponds to 20.8 h of benign and 17.2 h of spoofed audio data.

**SceneFake** (Yi et al., 2022b, 2024): This dataset focuses on detecting the manipulation of acoustic scenes, e.g., airport, street, or shopping, of audios. It consists of 19,838 real and 64,642 fake audio samples in English. The dataset was constructed based on ASVspoof 2019 LA (Wang et al., 2020) dataset and the acoustic scene dataset from DCASE2022^47^ challenge. The fake samples were generated by leveraging different speech enhancement models, including SSub, MMSE, Wiener, FullSubNet, WaveU-Net, and GCRN.

**Chinese fake audio detection dataset** (Ma et al., 2023a,b): This dataset consists of 115,800 real and 115,800 fake audio samples in Chinese. The audio samples in the dataset were generated by using the samples taken from 1302 speakers. The real audio samples were sourced from six different datasets, and twelve types of fake audio samples were generated from the real samples.

**EmoFake** (Zhao et al., 2023a,b): This dataset was designed for the detection of the changed emotion state of original audios. It comprises 35,000 real and 53,200 fake audio samples in English and supports five emotion states – *neutral, happy, sad, angry*, and *surprise*. The fake audio samples were generated by leveraging seven open-source emotion voice conversion models—VAW-GAN-CWT, DeepEST, Seq2Seq-EVC, CycleGAN-EVC, CycleTransGAN, EmoCycle-GAN, and StarGAN-EVC.

**PartialSpoof** (Zhang et al., 2021a,b): Similar to HAD, this dataset helps the detection of partially fake audio samples. It covers 12,483 real and 108,978 fake samples in English. The real samples in the dataset were collected from the ASVspoof 2019 LA dataset. Then, fake audios were generated by spoofing speech segments of the bona fide audio through voice conversion, text-to-speech, and audio splicing.

**ADD 2023 Dataset** (Yi et al., 2023): This is the dataset for the second Audio Deepfake Detection Challenge. The challenge had three main tracks – audio fake game (FG), manipulation region location (RL), and deepfake algorithm recognition (AR). In total, the dataset involves 243,194 real utterances and 273,874 fake utterances. The dataset contains utterances from the ADD 2022 Dataset as well as more fake audios generated by manipulating the original utterances with real or synthesised audios.

**Multi-language audio anti-spoofing dataset** (Müller et al., 2024): This dataset is based on MAILABS Speech Dataset and covers 163.9 h of synthetic voice. While the real video clips in the dataset involve human speeches in eight languages (i.e., English, French, German, Italian, Polish, Russian, Spanish, and Ukrainian), the dataset was enriched with computer-generated audio clips in 23 languages. For the generation of fake clips, 54 text-to-speech models comprising 21 different architectures were utilised.

### 4.4 Deepfake text datasets

Table 5 shows basic information about the text datasets covered.

**Table 5:** Deepfake-related text datasets.

**Dataset**	**Size**	**Year**	**Language**	**Generation method**
gpt-2-output-dataset	2,340,000 texts	2019	Not specified	GPT-2
TweepFake	25,572 tweets	2021	English	Fake account collection
TuringBench	168,612 news articles	2021	English	19 LLMs
GeneratedTextDetection	200 scientific papers + 200 abstracts	2022	English	GPT-2
ToxiGen	274,000 sentences	2022	English	GPT-3
MAGE	447,674 texts	2023	English	27 LLMs
AI-Writer	2,000 news articles	2023	Not specified	Text-generation service
ArticleForge	2,000 news articles	2023	Not specified	Text-generation service
Kafkai	2,000 articles	2023	Not specified	Text-generation service
RedditBot	1,774 comments	2023	Not specified	GPT-3 powered bot
IDMGSP	29,000 scientific papers	2023	English	Context-free grammar, LLMs
AIRABIC	1,000 texts	2023	Arabic	ChatGPT
HC3	125,230 answers to 37,175 questions	2023	English, Chinese	ChatGPT
Deepfake-BG	9,824 social media posts	2023	Bulgarian	GPT-2, ChatGPT
HC3 Plus	214,498 texts	2024	English, Chinese	ChatGPT
CHEAT	50,699 abstracts	2024	English	ChatGPT

**gpt-2-output-dataset** (OpenAI, 2019): This dataset contains outputs generated by GPT-2 trained on the OpenAI's Webtext training set. It comprises 250K training, 5K test, and 5K validation samples for each of the nine different GPT-2 models trained, in total, 2,340,000 text samples.

**TweepFake** (Fagni et al., 2021a,b): This dataset consists of 25,572 tweets collected from 23 bots, imitating 17 human accounts. The bots leveraged different methods for generating fake tweets—most were based on GPT-2 and RNN although other methods were also used, including Markov Chains and LSTM.

**TuringBench** (Uchendu et al., 2021): Focusing on the tasks of the Turing Test and Authorship Attribution for language models, this dataset is based on 10,000 news articles (mostly about politics). The titles of these articles were used to prompt 19 different LLMs (e.g., GPTs, GROVER, CTRL, XLM, PPLM etc.) to generate synthetic news articles similar to the original ones. After preprocessing, the size of the final dataset was reduced to 168,612.

**GeneratedTextDetection** (Liyanage et al., 2022a,b): This dataset contains two different corpora – 100 research papers collected from arXiv.org with 100 fully synthetic papers generated using GPT-2 and 100 original abstracts from arXiv.org preprints with 100 abstracts semi-automatically manipulated (i.e., keeping some sentences from the original content) using a GPT-2-based model, Arxiv-NLP.

**ToxiGen** (Hartvigsen et al., 2022): Being constructed for hate speech detection, this English dataset contains 137,000 benign and 137,000 toxic sentences, both generated using GPT-3. The sentences in the dataset mention 13 different minority groups without using explicit language.

**MAGE (formerly, DeepfakeTextDetect)** (Li et al., 2024): This dataset used different types of real texts collected from existing datasets, including opinion statements, news articles, answers to questions, stories, sentence sets for reasoning, Wikipedia paragraphs, and abstracts of scientific articles. Then, synthetic texts were generated using 27 LLMs with three different prompts. In total, it contains 447,674 human-written and machine-generated texts.

**AI-Writer** (Pu et al., 2023): This dataset utilised AI-Writer,^48^ i.e., an online commercial service for generating fake news articles, to generate 1,000 synthetic articles from 1000 real news titles sampled from the RealNews dataset.

**ArticleForge** (Pu et al., 2023): Similar to AI-Writer, this dataset was constructed using a commercial text generation service, ArticleForge,^49^ which requires a set of keywords to generate fake news articles. The dataset contains 1,000 real and 1,000 synthetic news articles.

**Kafkai** (Pu et al., 2023): This dataset leveraged the Kafkai commercial text generation service, which generates synthetic articles from a chosen category and an initial priming text. For the construction of the dataset, 100 real articles on 10 of the 25 available categories, such as cyber security, SEO, and marketing, were used to generate 1,000 fake articles.

**RedditBot** (Pu et al., 2023): This dataset is based on a GPT-3 powered bot posting comments on a popular subreddit on Reddit.com. 887 comments posted by the bot were collected as synthetic comments while the same amount of real comments were sampled randomly from the forum threads with a bot comment.

**Identifying machine-generated scientific papers** (Mosca et al., 2023): This dataset contains 16,000 real scientific papers retrieved from arXiv.org and 13,000 fake papers generated by utilising different methods. These methods involve LLMs, i.e., GPT-2, GPT-3, ChatGPT, and Galactica, as well as SCIgen which is based on context-free grammars. In total, the dataset consists of 25 million tokens.

**AIRABIC** (Alshammari and EI-Sayed, 2023a,b): This Arabic dataset includes 500 human-written texts and 500 ChatGPT-generated texts. The human-written texts were sourced from passages from books and news articles, covering both classical and modern standard Arabic. Moreover, the dataset contains different text variations, including single and multi-paragraph compositions, bullet points, and passages with in-text citations.

**Human ChatGPT comparison corpus** (Guo et al., 2023a,b): This bilingual (i.e., English and Chinese) dataset involves questions as well as answers to them provided by humans and ChatGPT to enable a comparison between human and ChatGPT answers to the same questions. In total, it covers 37,175 questions, 80,805 human answers, and 44,425 ChatGPT answers. The questions and human answers were collected from four English and six Chinese question-answering datasets. In addition, more question-answer pairs were constructed by crawling concept-explanation pairs from online encyclopaedias, Wikipedia^50^ and BaiduBaike.^51^

**Deepfake-BG** (Temnikova et al., 2023): This dataset consists of 4,912 human-written and 4,912 synthetic social media posts in Bulgarian. The human-written posts were randomly selected from multiple large datasets. The other posts were generated by leveraging two different LLMs—a new Bulgarian GPT-2 model, called GPT-WEB-BG, and ChatGPT for Bulgarian.

**HC3 Plus** (Su et al., 2024): This is an extended version of the HC3 dataset, covering translation, summarisation, and paraphrasing tasks. It contains 144,528 English texts and 69,970 Chinese texts, all generated by using ChatGPT. In terms of types of texts, the dataset involves news articles, social media posts, and questions.

**CHatGPT-writtEn AbsTract** (Yu et al., 2024a,b): This dataset contains 15,395 human-written abstracts and 35,304 ChatGPT-written abstracts. While human-written abstracts were collected from IEEE Xplore, ChatGPT was utilised to generate three types of synthetic abstracts—fully synthetic based on title and keywords, polished version of human-written abstracts, and a mixture of human-written abstracts and their polished versions.

### 4.5 Hybrid deepfake datasets

Table 6 shows basic information about the hybrid datasets covered.

**Table 6:** Hybrid deepfake-related datasets.

**Dataset**	**Size**	**Year**	**Language/Ethnicity**	**Generation method**
NIST open media forensics challenge datasets	Over 1,000 images and over 100 videos	2020	Not specified	GAN
ForgeryNet dataset	2,896,062 images and 221,247 videos	2021	Not Specified	Face reenactment, Face editing, Face transfer, Face swapping, Face stacked manipulation
Homologous deepfake dataset	6,802 images and 500 videos	2024	Chinese	Face swapping, Face reenactment, Attribute editing, GAN

**NIST OpenMFC (Open Media Forensics Challenge) Datasets**^52^: These datasets were created by the DARPA Media Forensics (MediFor) Program^53^ for the 2020 OpenMFC.^54^ There are two GAN-generated deepfake datasets, one with more than 1,000 deepfake images and the other with over 100 deepfake videos. The datasets were made available to registered participants of the competition only.

**ForgeryNet** (He et al., 2021): This dataset is named as “a versatile benchmark for comprehensive forgery analysis.” It contains 2,896,062 images and 221,247 videos, including 1,457,861 fake images and 121,617 fake videos. The videos and images cover seven image-level and eight video-level manipulation approaches, 36 different types of perturbations and more mixed perturbations, and a large number of annotation labels (6.3 million classification labels, 2.9 million manipulated area annotations and 221,247 temporal forgery segment labels). The manipulation approaches belong to five main generation methods—face reenactment, face editing, face transfer, face swapping, and face stacked manipulation. The dataset is being used for supporting the Face Forgery Analysis Challenge 2021^55^ at the SenseHuman 2021 (3rd Workshop on Sensing, Understanding and Synthesizing Humans),^56^ co-located at the ICCV 2021 conference.^57^

**Homologous deepfake dataset** (Xidian University, Modern Image Processing Lab, 2024): This dataset contains 6,802 fake images, 400 fake videos, and 100 real videos. According to the developers of the dataset, it is the first facial dataset for Chinese people. The dataset covers four types of deepfake multimedia, including full-face generation, attribute editing, face reenactment, and face swap.

### 4.6 Deepfake dataset generators

Despite not being datasets per se, dataset generators are systems for producing large datasets more automatically, including generating deepfake datasets. One may argue the automatically generated datasets are fake since they are not produced from real-world scenes. With this respect, we cover some state-of-the-art deepfake dataset generators that can be used to obtain new deepfake datasets, here.

**DatasetGAN** (Zhang Y. et al., 2021): This generator focuses on generating image-annotation pairs. It initially requires synthesising a small number of images with a GAN architecture and their annotation by humans. Then, an ensemble of multilayer perceptron (MLP) classifiers is trained on the pixel-wise feature vector of the used GAN architecture. This trained classifier is used as the label synthesis branch of the GAN architecture.

**BigDatasetGAN** (Li D. et al., 2022): This is an extended version of DatasetGAN, enhancing the ImageNET dataset, covering 1 million images, with pixel-wise labels. It leverages two ImageNET-pretrained models, BigGAN and VQGAN, to generate 5 samples for each of the 1,000 classes in ImageNET. Similar to DatasetGAN, these samples are manually labelled and used to train a classifier for pixel-level label synthesis.

**DatasetDM** (Wu et al., 2023): This aims to enhance the previous generators in terms of the produced image quality and generalisability. It makes use of text-to-image diffusion models (e.g., Stable Diffusion) to generate more realistic synthetic images with several perception annotations, including depth, segmentation, and human pose estimation. It also requires much fewer samples to be manually labelled, compared to the previous generators.

### 4.7 Subjective quality of deepfakes in different databases

As mentioned in Section 3.12, subjective quality evaluation is necessary to evaluate the realness, realisticness, and naturalness of deepfake media. While there has been very limited work on this topic, in 2020, Jiang et al. (2020) conducted a user study on the realness of deepfake videos. They recruited 100 professional participants (most of whom are computer vision researchers), who were asked to evaluate the realness of 30 randomly selected videos from 7 deepfake video datasets (DeeperForensics-1.0, UADFV, DeepFake-TIMIT, Celeb-DF, FaceForensics++, Deep Fake Detection, and DFDC). Participants were asked to respond to the statement “The video clip looks real.” and gave scores following a five-point Likert scale (1 – clearly disagree, 2 – weakly disagree, 3 – borderline, 4 – weakly agree, 5 – clearly agree). Table 7 shows the results. Interestingly, we can see a huge difference between the realness levels of different datasets. What is probably quite surprising is that FaceForensics++, one of the most widely used deepfake datasets, has a very low MOS score and less than 9% of participants considered the 30 selected videos as real.

**Table 7:** Human-judged subjective quality (realness) of deepfake videos in 7 datasets.

**Dataset**	**MOS**	**4+ ratings (%)**
DeeperForensics-1.0	3.806	64.1%
Celeb-DF	3.723	61.0%
DFDC	2.539	23%
Deep Fake Detection	2.518	21.9%
UADFV	2.249	14.1%
DeepFake-TIMIT	2.205	12.3%
FaceForensics++	1.874	8.4%

The MOS scores were not reported by Jiang et al. (2020), but calculated by us based on the raw data shown in Table 3 of Jiang et al. (2020).

### 4.8 Discussion: datasets

Among all deepfake image and video datasets, a significant majority are about face images and videos. This is not surprising since face swapping, face attribution manipulation, and fully synthesised face images are among the hottest topics within deepfake research and real-world applications. We hope more non-face deepfake image and video datasets can be produced to support a broader range of research activities on deepfake.

Several datasets mentioned in this survey overlooked reporting the language(s) and/or ethnicity(ies) they cover, which could be quite useful information in many applications. For those reporting the covered language(s), the majority focused on English, followed by Chinese. This indicates the need for new deepfake datasets covering underrepresented, and especially low-resource, languages and ethnicities.

The subjective quality results shown in Table 7 indicate that it is important to check the realness of deepfake media to support any performance evaluation or comparison. To ensure that the quality evaluation of datasets is fair, transparent and reliable, standard procedures need defining and a common pool of qualified human experts should be used.

Many authors of deepfake-related datasets attempted to classify such datasets into different generations. Chronologically speaking, we could broadly split such datasets into two generations: before 2019 and since 2019. Typically, datasets created before 2019 are relatively less advanced and smaller, while those created after 2019 tend to be larger, more diverse (i.e., covering more attributes), and of higher quality (i.e., produced by more advanced generative models). This can also be seen from the data in Table 7, in which the top two datasets (DeeperForensics-1 and Celeb-DF) fall within the new generation (2020), while others belong to the old generation. In addition to the two generations, a newer generation has also emerged in 2021: a number of very recent datasets started focusing on more realistic deepfakes (i.e., in the wild) or more specified areas of deepfakes (e.g., FFIW_10*K*_ focusing on multiple faces in the same video, and KoDF focusing on Korean faces). This trend shows that the deepfake research community has grown significantly in the past few years so narrower topics have also started gaining attention and interest from some researchers.

The introduction of conversational AI systems, especially ChatGPT, appeared as a game-changer for deepfake generation due to their high usability and accessibility. They have increasingly been used by researchers to generate deepfake datasets although their current usage is mostly limited to generating deepfake texts. However, we believe that new image, video, audio, and hybrid deepfake datasets can be constructed with such systems, considering the multimodal capabilities of the state-of-the-art generative AI models, e.g., GPT-4o.^58^

## 5 A meta-review of deepfake-related surveys

This section presents a meta-review of 15 selected deepfake-related survey papers published in English (Lyu, 2020; Tolosana et al., 2020; Tong et al., 2020; Verdoliva, 2020; Younus and Hasan, 2020; Zhang T. et al., 2020; Deshmukh and Wankhade, 2021; Mirsky and Lee, 2021; Nguyen et al., 2022; Rana et al., 2022; Seow et al., 2022; Heidari et al., 2023; Khanjani et al., 2023; Masood et al., 2023; Sandotra and Arora, 2024). It covers the following aspects in a systematic manner: definitions and scope, performance metrics, datasets, performance comparison, key challenges and recommendations.

The meta-review aims to draw some high-level insights for monitoring the future development of deepfake-related technologies and their applications.

### 5.1 Definitions and scope

As we discussed in Section 1.1, among researchers, practitioners and lawmakers there is no universally accepted definition of “deepfake” as a term. This is also reflected in how the authors of the 15 survey papers considered this aspect. Most authors talked about the history of deepfakes and pointed out that the term reflects the combination of “deep learning” and “fake,” but some used a broader definition, e.g., Lyu (2020) defined deepfake as “*high quality fake videos and audios generated by AI algorithms*.” Some authors also referred to deepfake-related legislations, but none of them pointed out that the definitions in some such legislations are completely different from the more technical definitions involving the use of deep learning. No authors discussed the blurred boundary between deepfakes and non-deepfakes.

In terms of the scope, while some authors (correctly) considered all types of media that can be produced by deepfake-related techniques (Lyu, 2020; Tong et al., 2020; Rana et al., 2022; Heidari et al., 2023; Masood et al., 2023; Sandotra and Arora, 2024), some considered only a narrow scope, e.g., authors of Tolosana et al. (2020), Younus and Hasan (2020), and Zhang T. et al. (2020) considered only videos, authors of Verdoliva (2020), Deshmukh and Wankhade (2021), Nguyen et al. (2022), and Seow et al. (2022) have only considered images and videos, and Khanjani et al. (2023) only considered audio deepfakes. Another phenomenon we observed is that many authors focused more on face images and videos, and authors of three surveys (Tolosana et al., 2020; Younus and Hasan, 2020; Deshmukh and Wankhade, 2021) even limited the definition of “deepfake” to such a narrow scope:

- Deshmukh and Wankhade (2021) defined it as “*a technology which creates fake images or videos of targeted humans by swapping their faces [by] another character saying or doing things that are not absolutely done by them and humans start believing in such fake as it is not always recognisable with the everyday human eye*;”
- Younus and Hasan (2020) considered deepfake as a technique allowing “*any computer user to exchange the face of one person with another digitally in any video*;” and
- Tolosana et al. (2020) defined it as “*a deep learning based technique able to create fake videos by swapping the face of a person by the face of another person*.”

Such unnecessarily narrow definitions and scopes can lead to confusion and do not help exchanges between researchers and practitioners working on different types of deepfakes.

We call on more researchers to accept a broader definition of “deepfake” so that highly realistic/natural media of any kind generated by a sophisticated automated method (often AI-based) is considered deepfake. Here, we provide two examples of such a broader definition: the image2image (or pixel2pixel) technique (Zhu et al., 2017) that allows the production of deepfake images and videos of any objects, and the so-called “deepfake geography (Zhao et al., 2021),” where AI-based techniques are used to generate realistic-looking satellite images.

Another important fact missed or not sufficiently discussed by authors of all the surveys, except Sandotra and Arora (2024), is that deepfake techniques can be used for positive applications, e.g., creative arts, entertainment and protecting online users' privacy. We call for more researchers and practitioners to follow the proposal in the 2020 Tencent AI White Paper (Tencent, 2020) to start using the more neutral-sounding term “deep synthesis.” Accordingly, we can use different words for different types of data generated using “deep synthesis” techniques, e.g., “deep art,” “deep animation,” “deep music,” and “deepfake.” While authors of most survey papers did not recognise the positive applications of “deepfake” technologies, Seow et al. (2022) and Sandotra and Arora (2024) covered positive applications, including entertainment, business, education, art, and medicine. Other than that, some other researchers also considered such applications, e.g., organisers of the Voice Conversion Challenge 2020 (see text footnote ^40^) who said the VC technology (for speech deepfake) “*is useful in many applications, such as customizing audio book and avatar voices, dubbing, movie industry, teleconferencing, singing voice modification, voice restoration after surgery, and cloning of voices of historical persons*.”

### 5.2 Performance metrics

Surprisingly, only two of the 15 surveys (Rana et al., 2022; Heidari et al., 2023) have covered performance metrics explicitly. Some directly used performance metrics to explain and compare the performance of covered deepfake generation and detection methods. The most used performance metrics include accuracy, ERR, and AUC. This may be explained by the page constraints of such survey papers, which did not allow the authors to extend their coverage significantly to cover performance metrics systematically. From this perspective, our Section 3 aims to fill this gap by providing a comprehensive coverage of relevant metrics and standards, including those specific to deepfakes. The subjective quality of deepfakes is an area least covered by the surveys, which seems related to an unbalanced coverage of deepfake generation and deepfake detection in terms of performance evaluation and comparison (the former much less than the latter).

### 5.3 Datasets

Many of the 15 survey papers list a number of deepfake-related datasets, but none of them has coverage as complete as ours shown in Section 4. Firstly, none of the surveys has covered text datasets, and only three of them (Heidari et al., 2023; Khanjani et al., 2023; Masood et al., 2023) mentioned audio datasets. When it comes to the coverage of image, video, and audio datasets, most surveys only listed more popular ones, instead of a more complete coverage of the available datasets. For instance, none of the surveys have covered the Voice Conversion Challenge 2016/2018/2020 datasets. In addition, more recent deepfake datasets especially those released since 2021 are also not covered by any of the surveys. We believe that our Section 4 is the most comprehensive review of deepfake-related datasets so far.

Some survey papers include datasets that are likely deepfakes, e.g., Verdoliva (2020) covered many general fake image datasets where the manipulated images were not generated by deep learning or even AI-based methods, and some surveys mentioned ASVspoof 2015 datasets but we did not see the use of deep learning for generating data used in the dataset.

### 5.4 Performance comparison

Most surveys have a good coverage of related methods for deepfake generation and detection, but only some explicitly covered performance comparison between different methods (Tolosana et al., 2020; Mirsky and Lee, 2021; Seow et al., 2022; Masood et al., 2023; Sandotra and Arora, 2024).

Due to quality issues of many deepfake-related datasets (discussed in Section 4.7), we need to treat any performance metrics and comparison of different detection methods with caution. Without testing all methods on a sufficiently large, diverse and high-quality deepfake dataset, the performance comparison results can be misleading. This highlights the importance of having more challenges, competitions and benchmarks to encourage performance comparison on standard datasets and using consistent performance metrics.

### 5.5 Challenges and recommendations

The authors of some surveys identified some key challenges and future research directions for the deepfake community.

Not surprisingly, how to develop more robust, scalable, generalisable and explainable deepfake detection methods is one of the most discussed key challenges and also a major future research direction (Lyu, 2020; Tong et al., 2020; Verdoliva, 2020; Younus and Hasan, 2020; Deshmukh and Wankhade, 2021; Rana et al., 2022; Heidari et al., 2023; Masood et al., 2023). Considering the arms race between deepfake generation and detection, this research direction will likely remain the hottest topic in deepfake research.

Some surveys (Verdoliva, 2020; Rana et al., 2022) mentioned fusion as a key future research direction, where “fusion” refers to combining different methods (e.g., combining multiple detectors of different types) and data sources (e.g., jointly considering audio-visual analysis) to achieve better performance for deepfake detection. Lyu (2020) suggested that, for the detection of deepfake videos, we need to consider video-level detection more, which can be considered fusion of detection results of all video frames.

The authors of many surveys (e.g., Lyu, 2020; Younus and Hasan, 2020; Deshmukh and Wankhade, 2021; Masood et al., 2023; Sandotra and Arora, 2024), argued that better (higher-quality, more up-to-date, and more standard) deepfake datasets are needed to develop more effective deepfake detection methods. Lyu (2020) and Masood et al. (2023) also suggested that we need to consider *social media laundering* effects in training data and improve the evaluation of datasets. We agree with them on these points. Finally, Rana et al. (2022) emphasised the differences in experimental settings of existing deepfake research and suggested a unique framework to be developed for the fair evaluation of deepfake detection methods.

There are also other *ad-hoc* recommendations given by the authors of some surveys. For example, Lyu (2020) argued that deepfake detection should be considered a (more complicated) multi-class, multi-label and local detection problem. Tolosana et al. (2020) discussed specific research directions for different deepfake generation methods (face synthesis, identity swap, attribute manipulation, and expression swap). Similarly, Heidari et al. (2023) and Masood et al. (2023) provided comprehensive discussions on future trends regarding the understanding, generation, detection, and prevention of deepfakes. Regarding preventing deepfakes, Heidari et al. (2023) and Khanjani et al. (2023) mentioned that blockchains and distributed ledger technologies can be leveraged for enhanced digital content traceability and identity sovereignty. Finally, Heidari et al. (2023) and Nguyen et al. (2022) underlined the importance of considering the human aspects of deepfake detection as well as the societal impacts of deepfakes, indicating the need for more interdisciplinary research on the subject.

## 6 Conclusion

The rapid growth in the capability to manipulate media or create synthetic media which look realistic and natural paved the way for deepfakes. At first, this paper adopted a critical approach to look at different definitions of the term “deepfake.” In that regard, we point out the different contradicting definitions and call for the wider community to consider how to define a new term that has a more consistent scope and meaning. For instance, replacing “deepfake” with “deep synthesis” can be more inclusive by embracing positive applications of deepfake techniques, e.g., in entertainment and for simulation purposes.

This paper provided a comprehensive overview of multiple aspects of the deepfake ecosystem drawing from the research literature and other online sources. It covers commonly used performance metrics, standards, and related datasets. It also presents a meta-review of 15 selected deepfake-related survey papers published since 2020, covering not only the above-mentioned aspects but also highlighting key challenges and recommendations.

## Footnotes

^1^ https://www.congress.gov/bill/115th-congress/senate-bill/3805

^2^ https://www.congress.gov/bill/116th-congress/house-bill/3230

^3^ https://www.iso.org/

^4^ https://www.iec.ch/

^5^ https://www.itu.int/

^6^ https://www.itu.int/rec/T-REC-P.800.1-201607-I/en

^7^ https://www.itu.int/rec/T-REC-P.910-200804-I/en

^8^ https://openai.com/index/dall-e/

^9^ https://www.iso.org/committee/6794475.html

^10^ http://www.iso.org/iso/jtc1_home.html

^11^ https://www.iso.org/standard/77609.html

^12^ https://www.iso.org/standard/79799.html

^13^ https://www.iso.org/standard/82570.html

^14^ https://www.iso.org/committee/313770.html

^15^ **https://www.iso.org/standard/73515.html**

^16^ https://www.iso.org/standard/53227.html

^17^ **https://www.iso.org/standard/67381.html**

^18^ https://sites.google.com/qq.com/face-anti-spoofing/

^19^ https://github.com/592McAvoy/fake-face-detection#user-content-i-dataset

^21^ https://github.com/media-sec-lab/Audio-Deepfake-Detection?tab=readme-ov-file#Datasets

^22^ https://github.com/MarekKowalski/FaceSwap/

^23^ https://generated.photos/datasets

^24^ https://generated.photos/face-generator/new

^25^ https://generated.photos/api

^26^According to a number of research papers we read, an earlier 100K-Faces dataset was released by generated.photos for academic research in 2018, which was used by many researchers. This dataset is not currently available any longer.

^27^Table 2 of the paper shows the dataset size is 19,509, but the dataset downloaded from pCloud contains just 19,457 images.

^28^ https://github.com/NVlabs/ffhq-dataset

^29^ https://github.com/fyu/lsun

^30^ https://drive.google.com/open?id=0B4qLcYyJmiz0TXY1NG02bzZVRGs

^31^ https://generated.photos/

^32^ http://mmlab.ie.cuhk.edu.hk/projects/CelebA.html

^33^ https://github.com/ondyari/FaceForensics

^34^ https://en.wikipedia.org/wiki/Nicolas_Cage

^35^ https://github.com/yuezunli/celeb-deepfakeforensics/tree/master/Celeb-DF-v1

^36^ https://ai.googleblog.com/

^37^ https://jigsaw.google.com/

^38^A Chinese video portal: https://www.bilibili.com/.

^39^ https://github.com/deepfakes/faceswap

^40^ http://www.vc-challenge.org/

^41^ http://www.vc-challenge.org/vcc2016/papers/SSW9_VCC2016_Results.pdf

^42^ https://www.emime.org/participate/emime-bilingual-database.html

^43^ https://www.asvspoof.org/

^44^ https://doi.org/10.7488/ds/1994

^45^ https://www.cs.toronto.edu/~graves/phd.pdf

^46^ https://github.com/syang1993/gst-tacotron

^47^ https://dcase.community/challenge2022/index

^48^ https://ai-writer.com/

^49^ https://www.articleforge.com/

^50^ https://www.wikipedia.org/

^51^ https://baike.baidu.com/

^52^ https://mfc.nist.gov/#pills-data

^53^ https://www.darpa.mil/program/media-forensics

^54^ https://mfc.nist.gov/

^55^ https://competitions.codalab.org/competitions/33386

^56^ https://sense-human.github.io/

^57^ http://iccv2021.thecvf.com/

^58^ https://openai.com/index/hello-gpt-4o/

## References

[B1] Afchar D. Nozick V. Yamagishi J. Echizen I. (2018). “MesoNet: a compact facial video forgery detection network,” in Proceedings of the 2018 IEEE International Workshop on Information Forensics and Security (IEEE), 1–7. 10.1109/WIFS.2018.8630761

[B2] Ajder H. Patrini G. Cavalli F. Cullen L. (2019). The state of deepfakes: Landscape, threats, and impact. Technical report, Deeptrace. Available at: https://sensity.ai/reports/ (accessed March, 2024)

[B3] Akhtar Z. Falk T. H. (2017). Audio-visual multimedia quality assessment: a comprehensive survey. IEEE Access 5, 21090–21117. 10.1109/ACCESS.2017.2750918

[B4] Alshammari H. EI-Sayed A. (2023b). AIRABIC: Arabic dataset for performance evaluation of ai detectors. GitHub dataset. Available at: https://github.com/Hamed1Hamed/AIRABIC (accessed July, 2024).

[B5] Alshammari H. Sayed A. (2023a). AIRABIC: Arabic dataset for performance evaluation of ai detectors,” in *Proceedings of the 2023 International Conference on Machine Learning and Applications (ICMLA)* (IEEE), 864–870. 10.1109/ICMLA58977.2023.00127

[B6] Ba Z. Wen Q. Cheng P. Wang Y. Lin F. Lu L.. (2023a). DEepfake CROss-lingual (DECRO) evaluation dataset. GitHub dataset. Available at: https://github.com/petrichorwq/DECRO-dataset (accessed March, 2024)

[B7] Ba Z. Wen Q. Cheng P. Wang Y. Lin F. Lu L.. (2023b). Transferring audio deepfake detection capability across languages,” in *Proceedings of the ACM Web Conference 2023* (ACM), 2033–2044. 10.1145/3543507.3583222

[B8] Bandi A. Adapa P. V. S. R. Kuchi Y. E. V. P. K. (2023). The power of generative AI: A review of requirements, models, input-output formats, evaluation metrics, and challenges. Fut. Internet 15:260. 10.3390/fi15080260

[B9] Brady M. (2020). Deepfakes: a new desinformation threat? Technical report, Democracy Reporting International. Available at: https://democracy-reporting.org/dri_publications/deepfakes-a-new-disinformation-threat/ (accessed March, 2024)

[B10] Cai Z. Ghosh S. Adatia A. P. Hayat M. Dhall A. Stefanov K. (2023a). AV-Deepfake1M: A large-scale LLM-driven audio-visual deepfake dataset. arXiv:2311.15308.

[B11] Cai Z. Ghosh S. Adatia A. P. Hayat M. Dhall A. Stefanov K. (2023b). AV-Deepfake1M: a large-scale LLM-driven audio-visual deepfake dataset. GitHub dataset. Available at: https://github.com/ControlNet/AV-Deepfake1M (accessed July, 2024).

[B12] Chefer H. Alaluf Y. Vinker Y. Wolf L. Cohen-Or D. (2023). Attend-and-Excite: attention-based semantic guidance for text-to-image diffusion models. ACM Trans. Graph. 42, 1–10. 10.1145/3592116

[B13] Chen Y. Liu L. Ding C. (2023). X-IQE: eXplainable image quality evaluation for text-to-image generation with visual large language models. arXiv:2305.10843.

[B14] Ciftci U. A. Demir I. Yin L. (2020). “FakeCatcher: Detection of synthetic portrait videos using biological signals,” in IEEE Transactions on Pattern Analysis and Machine Intelligence. 32750816 10.1109/TPAMI.2020.3009287

[B15] Dang H. Liu F. Stehouwer J. Liu X. Jain A. K. (2020). “On the detection of digital face manipulation,” in Proceedings of the 2020 IEEE/CVF Conference on Computer Vision and Pattern Recognition (IEEE), 5781–5790. 10.1109/CVPR42600.2020.00582

[B16] Delgado H. Evans N. Kinnunen T. Lee K. A. Liu X. Nautsch A.. (2021a). ASVspoof 2021 challenge - *logical access database*. Zenodo dataset.39215070

[B17] Delgado H. Evans N. Kinnunen T. Lee K. A. Liu X. Nautsch A.. (2021b). ASVspoof 2021 challenge - *speech deepfake database*. Zenodo dataset.39215070

[B18] Deng J. Dong W. Socher R. Li L.-J. Li K. Fei-Fei L. (2009). “ImageNet: a large-scale hierarchical image database,” in Proceedings of the 2009 IEEE Conference on Computer Vision and Pattern Recognition (IEEE), 248–255. 10.1109/CVPR.2009.5206848

[B19] Deshmukh A. Wankhade S. B. (2021). “Deepfake detection approaches using deep learning: A systematic review,” in Intelligent Computing and Networking: Proceedings of IC-ICN 2020, volume 146 of Lecture Notes in Networks and Systems (Springer), 293–302. 10.1007/978-981-15-7421-4_27

[B20] Ding X. Raziei Z. Larson E. C. Olinick E. V. Krueger P. Hahsler M. (2020). Swapped face detection using deep learning and subjective assessment. EURASIP J. Inf. Secur. 2020, 1–12. 10.1186/s13635-020-00109-8

[B21] Dolhansky B. Bitton J. Pflaum B. Lu J. Howes R. Wang M.. (2020). The DeepFake detection challenge dataset. arXiv preprint arXiv:2006.07397.

[B22] Dufour N. Gully A. (2019). Contributing data to deepfake detection research. Technical report, Google AI. Available at: https://ai.googleblog.com/2019/09/contributing-data-to-deepfake-detection.html (accessed March, 2024)

[B23] Durall R. Keuper M. Pfreundt F.-J. Keuper J. (2019). Unmasking deepfakes with simple features. arXiv:1911.00686.

[B24] Fagni T. Falchi F. Gambini M. Martella A. Tesconi M. (2021a). TweepFake: about detecting deepfake tweets. PLoS ONE 16:e0251415. 10.1371/journal.pone.0251415 33984021 PMC8118345

[B25] Fagni T. Falchi F. Gambini M. Martella A. Tesconi M. (2021b). TweepFake: about detecting deepfake tweets. GitHub dataset. Available at: https://github.com/tizfa/tweepfake_deepfake_text_detection (accessed July, 2024). 10.1371/journal.pone.0251415 PMC8118345 33984021

[B26] Fox G. Liu W. Kim H. Seidel H.-P. Elgharib M. Theobalt C. (2021). “Videoforensicshq: detecting high-quality manipulated face videos,” in Proceedings of the 2021 IEEE International Conference on Multimedia and Expo (IEEE), 1–6. 10.1109/ICME51207.2021.9428101

[B27] Frank J. Schönherr L. (2021a). “WaveFake: a data set to facilitate audio deepfake detection,” in Proceedings of the 35th Conference on Neural Information Processing Systems (NeurIPS 2021) Track on Datasets and Benchmarks, 1–17.

[B28] Frank J. Schönherr L. (2021b). WaveFake: a data set to facilitate audio deepfake detection. GitHub dataset. Available at: https://github.com/RUB-SysSec/WaveFake

[B29] Gong Y. Yang J. Huber J. MacKnight M. Poellabauer C. (2019a). ReMASC: realistic replay attack corpus for voice controlled systems,” in Proceedings of Interspeech 2019, 2355–2359. 10.21437/Interspeech.2019-1541

[B30] Gong Y. Yang J. Huber J. MacKnight M. Poellabauer C. (2019b). ReMASC: realistic replay attack corpus for voice controlled systems. GitHub dataset. Available at: https://github.com/YuanGongND/ReMASC (accessed July, 2024).

[B31] Guo B. Zhang X. Wang Z. Jiang M. Nie J. Ding Y.. (2023a). How close is ChatGPT to human experts? comparison corpus, evaluation, and detection. arXiv:2301.07597.

[B32] Guo B. Zhang X. Wang Z. Jiang M. Nie J. Ding Y.. (2023b). Human ChatGPT Comparison Corpus (HC3). GitHub dataset. Available at: https://github.com/Hello-SimpleAI/chatgpt-comparison-detection (accessed July, 2024)

[B33] Hartvigsen T. Gabriel S. Palangi H. Sap M. Ray D. Kamar E. (2022). “ToxiGen: a large-scale machine-generated dataset for adversarial and implicit hate speech detection,” in Proceedings of the 60th Annual Meeting of the Association for Computational Linguistics (ACL), 3309–3326. 10.18653/v1/2022.acl-long.234

[B34] He Y. Gan B. Chen S. Zhou Y. Yin G. Song L.. (2021). “ForgeryNet: a versatile benchmark for comprehensive forgery analysis,” in Proceedings of the 2021 IEEE/CVF Conference on Computer Vision and Pattern Recognition (IEEE), 4360–4369. 10.1109/CVPR46437.2021.00434

[B35] Heidari A. Jafari Navimipour N. Dag H. Unal M. (2023). Deepfake detection using deep learning methods: a systematic and comprehensive review. WIREs Data Mining Knowl. Discov. 45:e1520. 10.1002/widm.152034460519

[B36] Hessel J. Holtzman A. Forbes M. Le Bras R. Choi Y. (2021). “CLIPScore: a reference-free evaluation metric for image captioning,” in Proceedings of the 2021 Conference on Empirical Methods in Natural Language Processing (Association for Computational Linguistics), 7514–7528. 10.18653/v1/2021.emnlp-main.595

[B37] Huang K. Sun K. Xie E. Li Z. Liu X. (2023). “T2I-CompBench: a comprehensive benchmark for open-world compositional text-to-image generation,” in Proceedings of the 37th Neural Information Processing Systems Track on Datasets and Benchmarks (NeurIPS '23) (Curran Associates, Inc.), 78723–78747.

[B38] Jia S. Li X. Lyu S. (2022a). DFDM: Deepfakes from different models. GitHub dataset. Available at: https://github.com/shanface33/Deepfake_Model_Attribution (accessed March, 2024)

[B39] Jia S. Li X. Lyu S. (2022b). Model attribution of face-swap deepfake videos. arXiv:2202.12951. 10.1109/ICIP46576.2022.9897972

[B40] Jiang L. Li R. Wu W. Qian C. Loy C. C. (2020). “DeeperForensics-1.0: a large-scale dataset for real-world face forgery detection,” in Proceedings of the 2020 IEEE/CVF Conference on Computer Vision and Pattern Recognition (IEEE), 2886–2895. 10.1109/CVPR42600.2020.00296

[B41] Kalchbrenner N. Elsen E. Simonyan K. Noury S. Casagrande N. Lockhart E.. (2018). Efficient neural audio synthesis. arXiv:1802.08435.

[B42] Karras T. Laine S. Aila T. (2019). “A style-based generator architecture for generative adversarial networks,” in Proceedings of the 2019 IEEE/CVF Conference on Computer Vision and Pattern Recognition (IEEE), 4401–4410. 10.1109/CVPR.2019.00453 32012000

[B43] Khanjani Z. Watson G. Janeja V. P. (2023). Audio deepfakes: a survey. Front. Big Data 5:1001063. 10.3389/fdata.2022.1001063 36700137 PMC9869423

[B44] Khodabakhsh A. Ramachandra R. Raja K. Wasnik P. Busch C. (2018). “Fake face detection methods: can they be generalized?” in Proceedings of the 2018 International Conference of the Biometrics Special Interest Group (IEEE), 1–6. 10.23919/BIOSIG.2018.8553251

[B45] Kim H. Elgharib M. Zollhöfer M. Seidel H. P. Beeler T. Richardt C.. (2019). Neural style-preserving visual dubbing. ACM Trans. Graph. 38, 1–13. 10.1145/3355089.3356500

[B46] Kim H. Garrido P. Tewari A. Xu W. Thies J. Niessner M.. (2018). Deep video portraits. ACM Trans. Graph. 37, 1–14. 10.1145/3197517.320128327187945

[B47] Korshunov P. Marcel S. (2019). “Vulnerability assessment and detection of deepfake videos,” in Proceedings of the 2019 International Conference on Biometrics (IEEE), 1–6. 10.1109/ICB45273.2019.8987375

[B48] Kwon P. You J. Nam G. Park S. Chae G. (2021). “KoDF: A large-scale korean DeepFake detection dataset,” in Proceedings of the 2021 IEEE/CVF International Conference on Computer Vision (IEEE), 10724–10733. 10.1109/ICCV48922.2021.01057

[B49] Li D. Ling H. Kim S. W. Kreis K. Fidler S. Torralba A. (2022). “BigDatasetGAN: Synthesizing imagenet with pixel-wise annotations,” in Proceedings of the IEEE/CVF Conference on Computer Vision and Pattern Recognition (CVPR) (IEEE), 21330–21340. 10.1109/CVPR52688.2022.02064

[B50] Li G. Zhao X. Cao Y. Pei P. Li J. Zhang Z. (2022a). “FMFCC-V: an Asian large-scale challenging dataset for deepfake detection,” in Proceedings of the 2022 ACM Workshop on Information Hiding and Multimedia Security (ACM), 7–18. 10.1145/3531536.3532946

[B51] Li G. Zhao X. Cao Y. Pei P. Li J. Zhang Z. (2022b). FMFCC-V: an Asian large-scale challenging dataset for deepfake detection. GitHub dataset. Available at: https://github.com/iiecasligen/FMFCC-V (accessed July, 2024).

[B52] Li J. Li D. Xiong C. Hoi S. (2022). “BLIP: bootstrapping language-image pre-training for unified vision-language understanding and generation,” in Proceedings of the 39th International Conference on Machine Learning (PMLR), 12888–12900.

[B53] Li L. Bao J. Yang H. Chen D. Wen F. (2020). “Advancing high fidelity identity swapping for forgery detection,” in Proceedings of the 2020 IEEE/CVF Conference on Computer Vision and Pattern Recognition, 5073–5082. 10.1109/CVPR42600.2020.00512

[B54] Li Y. Chang M.-C. Lyu S. (2018). “In ICTU OCULI: exposing AI created fake videos by detecting eye blinking,” in Proceedings of the 2018 IEEE International Workshop on Information Forensics and Security (IEEE), 1–7. 10.1109/WIFS.2018.8630787

[B55] Li Y. Li Q. Cui L. Bi W. Wang Z. Wang L.. (2024). MAGE: machine-generated text detection in the wild. arXiv:2305.13242.

[B56] Li Y. Yang X. Sun P. Qi H. Lyu S. (2020). “Celeb-DF: a large-scale challenging dataset for deepfake forensics,” in Proceedings of the 2020 IEEE/CVF Conference on Computer Vision and Pattern Recognition (IEEE), 3204–3213. 10.1109/CVPR42600.2020.00327

[B57] Liu X. Wang X. Sahidullah M. Patino J. Delgado H. Kinnunen T.. (2023). ASVspoof 2021: towards spoofed and deepfake speech detection in the wild. IEEE/ACM Trans. Audio, Speech Lang. Proc. 31, 2507–2522. 10.1109/TASLP.2023.3285283

[B58] Livingstone S. R. Russo F. A. (2018). The ryerson audio-visual database of emotional speech and song (RAVDESS): a dynamic, multimodal set of facial and vocal expressions in north american english. PLoS ONE 13:e0196391. 10.1371/journal.pone.0196391 29768426 PMC5955500

[B59] Liyanage V. Buscaldi D. Nazarenko A. (2022a). A benchmark corpus for the detection of automatically generated text in academic publications. arXiv:2202.02013.

[B60] Liyanage V. Buscaldi D. Nazarenko A. (2022b). GeneratedTextDetection. GitHub dataset. Available at: https://github.com/vijini/GeneratedTextDetection (accessed July, 2024)

[B61] Lorenzo-Trueba J. Yamagishi J. Toda T. Saito D. Villavicencio F. Kinnunen T.. (2018). “The voice conversion challenge 2018: Promoting development of parallel and nonparallel methods,” in Proceedings of the Odyssey 2018 The Speaker and Language Recognition Workshop (International Speech Communication Association), 195–202. 10.21437/Odyssey.2018-28

[B62] Lyu S. (2020). “Deepfake detection: Current challenges and next steps,” in Proceedings of the 2020 IEEE International Conference on Multimedia Expo Workshops (IEEE) 10.1109/ICMEW46912.2020.9105991

[B63] Ma H. Yi J. Wang C. Yan X. Tao J. Wang T.. (2023a). CFAD: a Chinese dataset for fake audio detection. arXiv:2207.12308. 10.2139/ssrn.4748856

[B64] Ma H. Yi J. Wang C. Yan X. Tao J. Wang T.. (2023b). CFAD: a Chinese dataset for fake audio detection. GitHub dataset. Available at: https://github.com/ADDchallenge/CFAD (accessed July, 2024).

[B65] Masood M. Nawaz M. Malik K. M. Javed A. Irtaza A. Malik H. (2023). Deepfakes generation and detection: State-of-the-art, open challenges, countermeasures, and way forward. Appl. Intell. 53, 3974–4026. 10.1007/s10489-022-03766-z

[B66] Mirsky Y. Lee W. (2021). The creation and detection of deepfakes: a survey. ACM Comput. Surv. 54, 1–41. 10.1145/3425780

[B67] Mosca E. Abdalla M. H. I. Basso P. Musumeci M. Groh G. (2023). “Distinguishing fact from fiction: a benchmark dataset for identifying machine-generated scientific papers in the LLM era,” in Proceedings of the 3rd Workshop on Trustworthy Natural Language Processing (TrustNLP 2023) (ACL), 190–207. 10.18653/v1/2023.trustnlp-1.17

[B68] Müller N. Czempin P. Diekmann F. Froghyar A. Böttinger K. (2022). “Does audio deepfake detection generalize?,” in Proceedings of Interspeech 2022, 2783–2787. 10.21437/Interspeech.2022-108

[B69] Müller N. M. Kawa P. Choong W. H. Casanova E. Gölge E. Müller T.. (2024). MLAAD: The multi-language audio anti-spoofing dataset. arXiv:2401.09512.

[B70] Mysore G. J. (2015). Can we automatically transform speech recorded on common consumer devices in real-world environments into professional production quality speech?–a dataset, insights, and challenges. IEEE Signal Proc. Lett. 22, 1006–1010. 10.1109/LSP.2014.2379648

[B71] Narayan K. Agarwal H. Thakral K. Mittal S. Vatsa M. Singh R. (2023a). Df-Platter database. Public dataset. Available at: https://iab-rubric.org/df-platter-database (accessed March, 2024)

[B72] Narayan K. Agarwal H. Thakral K. Mittal S. Vatsa M. Singh R. (2023b). DF-Platter: multi-face heterogeneous deepfake dataset,” in *Proceedings of the 2023 IEEE/CVF Conference on Computer Vision and Pattern Recognition* (IEEE), 9739–9748. 10.1109/CVPR52729.2023.00939

[B73] Neves J. C. Tolosana R. Vera-Rodriguez R. Lopes V. Proena H. Fierrez J. (2020). GANprintR: improved fakes and evaluation of the state of the art in face manipulation detection. IEEE J. Select. Topics Signal Proc. 14, 1038–1048. 10.1109/JSTSP.2020.3007250

[B74] Nguyen T. T. Nguyen Q. V. H. Nguyen D. T. Nguyen D. T. Huynh-The T. Nahavandi S. . (2022). Deep learning for deepfakes creation and detection: a survey. Comput. Vis. Image Understand. 223:103525. 10.1016/j.cviu.2022.103525

[B75] Ni B. Peng H. Chen M. Zhang S. Meng G. Fu J.. (2022). “Expanding language-image pretrained models for general video recognition,” in Proceedings of the 17th European Conference on Computer Vision (ECCV '22) (Springer Nature Switzerland), 1–18. 10.1007/978-3-031-19772-7_1

[B76] Nirkin Y. Keller Y. Hassner T. (2019). “FSGAN: subject agnostic face swapping and reenactment,” in Proceedings of the 2019 IEEE/CVF International Conference on Computer Vision (IEEE), 7183–7192. 10.1109/ICCV.2019.00728

[B77] OpenAI (2019). GPT-2-output-dataset: dataset of GPT-2 outputs for research in detection, biases, and more. GitHub dataset. Available at: https://github.com/openai/gpt-2-output-dataset/ (accessed March, 2024)

[B78] Pal D. Triyason T. (2018). A survey of standardized approaches towards the quality of experience evaluation for video services: an ITU perspective. Int. J. Dig. Multimedia Broadcast. 2018:1391724. 10.1155/2018/1391724

[B79] Pu J. Mangaokar N. Kelly L. Bhattacharya P. Sundaram K. Javed M.. (2021a). “Deepfake videos in the wild: analysis and detection,” in Proceedings of the Web Conference 2021 (ACM), 981–992. 10.1145/3442381.3449978

[B80] Pu J. Mangaokar N. Kelly L. Bhattacharya P. Sundaram K. Javed M.. (2021b). DF-W: a new deepfake dataset comprising of deepfake videos created and shared by the internet community. GitHub dataset. Available at: https://github.com/jmpu/webconf21-deepfakes-in-the-wild (accessed March, 2024)

[B81] Pu J. Sarwar Z. Abdullah S. M. Rehman A. Kim Y. Bhattacharya P.. (2023). “Deepfake text detection: Limitations and opportunities,” in Proceedings of the 2023 IEEE Symposium on Security and Privacy (SP) (IEEE), 1613–1630. 10.1109/SP46215.2023.10179387

[B82] Radford A. Kim J. W. Hallacy C. Ramesh A. Goh G. Agarwal S.. (2021). “Learning transferable visual models from natural language supervision,” in Proceedings of the 38th International Conference on Machine Learning (PMLR), 8748–8763.

[B83] Rana M. S. Nobi M. N. Murali B. Sung A. H. (2022). Deepfake detection: a systematic literature review. IEEE Access 10, 25494–25513. 10.1109/ACCESS.2022.315440434460519

[B84] Rössler A. Cozzolino D. Verdoliva L. Riess C. Thies J. NieSSner M. (2018). FaceForensics: a large-scale video dataset for forgery detection in human faces. arXiv preprint arXiv:1803.09179.

[B85] Rössler A. Cozzolino D. Verdoliva L. Riess C. Thies J. NieSSner M. (2019). “FaceForensics++: learning to detect manipulated facial images,” in Proceedings of the 2019 International Conference on Computer Vision (IEEE), 1–11. 10.1109/ICCV.2019.00009

[B86] Rothe R. Timofte R. Van Gool L. (2015). “DEX: deep expectation of apparent age from a single image,” in Proceedings of the 2015 IEEE International Conference on Computer Vision Workshop (IEEE), 252–257. 10.1109/ICCVW.2015.41

[B87] Sandotra N. Arora B. (2024). A comprehensive evaluation of feature-based AI techniques for deepfake detection. Neural Comput. Applic. 36, 3859–3887. 10.1007/s00521-023-09288-0

[B88] Sensity (2024). The state of deepfakes 2024. Technical report, Sensity. Available at: https://sensity.ai/reports/ (accessed July, 2024)

[B89] Seow J. W. Lim M. K. Phan R. C. Liu J. K. (2022). A comprehensive overview of Deepfake: generation, detection, datasets, and opportunities. Neurocomputing 513, 351–371. 10.1016/j.neucom.2022.09.135

[B90] Song H. Huang S. Dong Y. Tu W.-W. (2023a). DeepFakeFace. GitHub dataset. Available at: https://github.com/OpenRL-Lab/DeepFakeFace (accessed March, 2024)

[B91] Song H. Huang S. Dong Y. Tu W.-W. (2023b). Robustness and generalizability of deepfake detection: a study with diffusion models. arXiv:2309.02218.

[B92] Su Z. Li M. Zhang G. Wu Q. Li M. Zhang W.. (2023a). CMFD. GitHub dataset. Available at: https://github.com/WuQinfang/CMFD (accessed March, 2024)

[B93] Su Z. Li M. Zhang G. Wu Q. Li M. Zhang W.. (2023b). Robust audio copy-move forgery detection using constant q spectral sketches and GA-SVM. IEEE Trans. Depend. Secure Comput. 20, 4016–4031. 10.1109/TDSC.2022.3215280

[B94] Su Z. Wu X. Zhou W. Ma G. Hu S. (2024). HC3 Plus: a semantic-invariant human ChatGPT comparison corpus. arXiv:2309.02731.

[B95] Tanaka K. Kameoka H. Kaneko T. Hojo N. (2019). WaveCycleGAN2: time-domain neural post-filter for speech waveform generation. arXiv:1904.02892.

[B96] Temnikova I. Marinova I. Gargova S. Margova R. Koychev I. (2023). “Looking for traces of textual deepfakes in Bulgarian on social media,” in Proceedings of the 14th International Conference on Recent Advances in Natural Language Processing (Shoumen, Bulgaria: INCOMA Ltd.), 1151–1161. 10.26615/978-954-452-092-2_122

[B97] Tencent (2020). Artificial intelligence white paper. Technical report, Tencent. Available at: https://tech.sina.com.cn/roll/2020-07-14/doc-iivhvpwx5201226.shtml (accessed March, 2024)

[B98] Toda T. Chen L.-H. Saito D. Villavicencio F. Wester M. Wu Z.. (2016). “The voice conversion challenge 2016,” in Proceedings of Interspeech 2016 (International Speech Communication Association), 1632–1636. 10.21437/Interspeech.2016-1066

[B99] Tolosana R. Vera-Rodriguez R. Fierrez J. Morales A. Ortega-Garcia J. (2020). Deepfakes and beyond: a survey of face manipulation and fake detection. Inf. Fusion 64, 131–148. 10.1016/j.inffus.2020.06.014

[B100] Tong X. Wang L. Pan X. Wang J. (2020). “An overview of deepfake: the sword of Damocles in AI,” in Proceedings of the 2020 International Conference on Computer Vision, Image and Deep Learning (IEEE), 265–273. 10.1109/CVIDL51233.2020.00-88

[B101] Uchendu A. Ma Z. Le T. Zhang R. Lee D. (2021). “TURINGBENCH: a benchmark environment for Turing test in the age of neural text generation,” in Findings of the Association for Computational Linguistics: EMNLP 2021 (ACL), 2001–2016. 10.18653/v1/2021.findings-emnlp.172

[B102] Van Den Oord A. Dieleman S. Zen H. Simonyan K. Vinyals O. Graves A.. (2016). WaveNet: a generative model for raw audio. arXiv:1609.03499.

[B103] Verdoliva L. (2020). Media forensics and deepfakes: an overview. IEEE J. Selected Topics Signal Proc. 14, 910–932. 10.1109/JSTSP.2020.300210138855214

[B104] Wang X. Yamagishi J. Todisco M. Delgado H. Nautsch A. Evans N.. (2020). ASVspoof 2019: A large-scale public database of synthesized, converted and replayed speech. Comput. Speech Lang.64:101114. 10.1016/j.csl.2020.101114

[B105] Wang Z. Bao J. Zhou W. Wang W. Hu H. Chen H.. (2023a). “DIRE for diffusion-generated image detection,” in Proceedings of the 2023 IEEE/CVF International Conference on Computer Vision (IEEE), 22388–22398. 10.1109/ICCV51070.2023.02051

[B106] Wang Z. Bao J. Zhou W. Wang W. Hu H. Chen H.. (2023b). DIRE for diffusion-generated image detection. GitHub dataset. Available at: https://github.com/ZhendongWang6/DIRE (accessed July, 2024).

[B107] Wei J. Wang X. Schuurmans D. Bosma M. Ichter B. Xia F.. (2022). “Chain-of-Thought prompting elicits reasoning in large language models,” in Proceedings of the 36th Neural Information Processing Systems (NeurIPS '22) (Curran Associates, Inc.), 24824–24837.

[B108] Wu J. Z. Fang G. Wu H. Wang X. Ge Y. Cun X.. (2024). Towards a better metric for text-to-video generation. arXiv:2401.07781.

[B109] Wu W. Zhao Y. Chen H. Gu Y. Zhao R. He Y.. (2023). “DatasetDM: synthesizing data with perception annotations using diffusion models,” in Proceedings of the 37th International Conference on Neural Information Processing Systems (Curran Associates, Inc.), 54683–54695.

[B110] Xidian University Modern Image Processing Lab. (2024). Homologous deepfake dataset: A self built small-scale, high-quality, and diverse deepfake dataset. GitHub dataset. Available at: https://github.com/mirro-yyf/Homologous_deepfake_dataset (accessed July, 2024)

[B111] Xie Y. Zhou J. Lu X. Jiang Z. Yang Y. Cheng H.. (2023a). FSD: An initial chinese dataset for fake song detection. GitHub dataset. Available at: https://github.com/xieyuankun/FSD-Dataset (accessed July, 2024)

[B112] Xie Y. Zhou J. Lu X. Jiang Z. Yang Y. Cheng H.. (2023b). FSD: an initial chinese dataset for fake song detection. GitHub dataset. Available at: https://github.com/xieyuankun/FSD-Dataset (accessed July, 2024).

[B113] Yariv G. Gat I. Benaim S. Wolf L. Schwartz I. Adi Y. (2024). “Diverse and aligned audio-to-video generation via text-to-video model adaptation,” in Proceedings of the 38th AAAI Conference on Artificial Intelligence (AAAI '24) (AAAI), 6639–6647. 10.1609/aaai.v38i7.28486

[B114] Yi J. Bai Y. Tao J. Ma H. Tian Z. Wang C.. (2021). “Half-Truth: a partially fake audio detection dataset,” in Proceedings of Interspeech 2021, 1654–1658.

[B115] Yi J. Fu R. Tao J. Nie S. Ma H. Wang C.. (2022a). “ADD 2022: the first audio deep synthesis detection challenge,” in Proceedings of the 2022 IEEE International Conference on Acoustics, Speech and Signal Processing (ICASSP) (IEEE), 9216–9220.

[B116] Yi J. Tao J. Fu R. Yan X. Wang C. Wang T.. (2023). “ADD 2023: the second audio deepfake detection challenge,” in Proceedings of the Workshop on Deepfake Audio Detection and Analysis (CEUR Workshop Proceedings), 125–130.

[B117] Yi J. Wang C. Tao J. Tian Z. Fan C. Ma H.. (2022b). SceneFake: an initial dataset and benchmarks for scene fake audio detection. GitHub dataset. Available at: https://github.com/ADDchallenge/SceneFake (accessed July, 2024)

[B118] Yi J. Wang C. Tao J. Zhang C. Y. Fan C. Tian Z.. (2024). SceneFake: an initial dataset and benchmarks for scene fake audio detection. Patt. Recogn. 152:110468. 10.1016/j.patcog.2024.110468

[B119] Yi Z. Huang W.-C. Tian X. Yamagishi J. Das R. K. Kinnunen T.. (2020). “Voice conversion challenge 2020-intra-lingual semi-parallel and cross-lingual voice conversion.,” in Proceedings of the Joint Workshop for the Blizzard Challenge and Voice Conversion Challenge 2020 (International Speech Communication Association), 80–98. 10.21437/VCCBC.2020-14

[B120] Younus M. A. Hasan T. M. (2020). “Abbreviated view of deepfake videos detection techniques,” in Proceedings of the 2020 6th International Engineering Conference (IEEE), 115–120. 10.1109/IEC49899.2020.9122916

[B121] Yu P. Chen J. Feng X. Xia Z. (2024a). CHEAT. GitHub dataset. Available at: https://github.com/botianzhe/CHEAT (accessed July, 2024)

[B122] Yu P. Chen J. Feng X. Xia Z. (2024b). CHEAT: A large-scale dataset for detecting ChatGPT-written abstracts. arXiv:2304.12008.

[B123] Zhai G. Min X. (2020). Perceptual image quality assessment: a survey. Sci. China Inf. Sci. 63, 1–52. 10.1007/s11432-019-2757-1

[B124] Zhang L. Wang X. Cooper E. Yamagishi J. Patino J. Evans N. (2021a). “An initial investigation for detecting partially spoofed audio,” in Proceedings of Interspeech 2021, 4264–4268. 10.21437/Interspeech.2021-738

[B125] Zhang L. Wang X. Cooper E. Yamagishi J. Patino J. Evans N. (2021b). PartialSpoof . Zenodo dataset. Available at: https://zenodo.org/records/5766198 (accessed March, 2024)

[B126] Zhang T. Deng L. Zhang L. Dang X. (2020). “Deep learning in face synthesis: a survey on deepfakes,” in Proceedings of the 2020 IEEE 3rd International Conference on Computer and Communication Engineering Technology (IEEE), 67–70. 10.1109/CCET50901.2020.9213159

[B127] Zhang Y. Ling H. Gao J. Yin K. Lafleche J.-F. Barriuso A.. (2021). “DatasetGAN: Efficient labeled data factory with minimal human effort,” in Proceedings of the 2021 IEEE/CVF Conference on Computer Vision and Pattern Recognition (IEEE), 10140–10150. 10.1109/CVPR46437.2021.01001

[B128] Zhang Y. Yin Z. Li Y. Yin G. Yan J. Shao J.. (2020). “CelebA-Spoof: large-scale face anti-spoofing dataset with rich annotations,” in Proceedings of the 2020 European Conference on Computer Vision (Springer), 70–85. 10.1007/978-3-030-58610-2_5

[B129] Zhao B. Zhang S. Xu C. Sun Y. Deng C. (2021). Deep fake geography? When geospatial data encounter artificial intelligence. Cartogr. Geogr. Inf. Sci. 48, 338–352. 10.1080/15230406.2021.1910075

[B130] Zhao Y. Yi J. Tao J. Wang C. Zhang X. Dong Y. (2023a). EmoFake: an initial dataset for emotion fake audio detection. arXiv:2211.05363.

[B131] Zhao Y. Yi J. Tao J. Wang C. Zhang X. Dong Y. (2023b). EmoFake: an initial dataset for emotion fake audio detection. GitHub dataset. Available at: https://github.com/zy511361103/GADE (accessed July, 2024).

[B132] Zhou P. Han X. Morariu V. I. Davis L. S. (2017). “Two-stream neural networks for tampered face detection,” in Proceedings of the 2017 IEEE Conference on Computer Vision and Pattern Recognition Workshops (IEEE), 1831–1839. 10.1109/CVPRW.2017.229

[B133] Zhou T. Wang W. Liang Z. Shen J. (2021). “Face forensics in the wild,” in Proceedings of the 2021 IEEE/CVF Conference on Computer Vision and Pattern Recognition (IEEE), 5774–5784. 10.1109/CVPR46437.2021.00572

[B134] Zhu D. Chen J. Shen X. Li X. Elhoseiny M. (2024). “MiniGPT-4: enhancing vision-language understanding with advanced large language models,” in Proceedings of the 12th International Conference on Learning Representations.38329788

[B135] Zhu J.-Y. Park T. Isola P. Efros A. A. (2017). “Unpaired image-to-image translation using cycle-consistent adversarial networks,” in Proceedings of the 2017 IEEE International Conference on Computer Vision (IEEE), 2242–2251. 10.1109/ICCV.2017.244

[B136] Zi B. Chang M. Chen J. Ma X. Jiang Y.-G. (2020). “WildDeepfake: a challenging real-world dataset for deepfake detection,” in Proceedings of the 2020 28th ACM International Conference on Multimedia (ACM), 2382–2390.

